# Advances in the characterisation and identification of mastic (*Pistacia* sp.) resin in archaeological samples by GC-QToF-MS†

**DOI:** 10.1039/d3ra06651g

**Published:** 2024-01-02

**Authors:** Diego Tamburini, Kate Fulcher, Lisa Briggs, Nelly von Aderkas, Cemal Pulak, Rebecca Stacey

**Affiliations:** a Department of Scientific Research, The British Museum Great Russell Street London WC1B 3DG UK DTamburini@britishmuseum.org; b Department of Anthropology, Institute of Nautical Archaeology at Texas A&M University, Texas A&M University College Station Texas USA

## Abstract

The optimisation and application of an analytical method based on gas chromatography coupled to quadrupole time-of-flight mass spectrometry (GC-QToF-MS) is proposed for the first time for the characterisation and identification of mastic (*Pistacia* sp.) resin in archaeological samples. The GC-QToF-MS method demonstrated higher sensitivity compared to single quadrupole GC-MS and enabled enhanced structural elucidation power to be exploited, particularly due to the high mass resolution and accuracy, the possibility to use standard and low ionisation energies as well as its tandem MS capabilities. The heat-induced degradation of the resin was also studied in open air conditions, showing that 28-norolean-17-en-3-one forms upon heating, but then progressively degrades. This makes it a reliable marker for heating of *Pistacia* resin; however, the lack of detection does not imply that the resin was not heated. These observations were used to interpret the results of a large number of archaeological samples containing *Pistacia* resin in different formulations, from various archaeological contexts and exposed to different environmental conditions. Lumps of relatively pure resin found in marine waterlogged conditions (Uluburun shipwreck, Turkey), residues on ceramics from Sai Island (Nubia, Sudan) as well as varnish and coating layers on Egyptian coffins from the collections of the British Museum (London, UK) and Fitzwilliam Museum (Cambridge, UK) were analysed to understand what the molecular profiles reveal about the use of the resin. The results showed that the resin was often mixed with a drying or semi-drying oil in ancient varnish formulations, thus suggesting that oil was used as a medium to dissolve the resin, which would have been impossible to apply as a layer using simple heat. These new observations significantly add to our understanding of ancient Egyptian technology and provide museum scientists and conservators with key information to accurately identify *Pistacia* resin and preserve objects containing it.

## Abstract 2

A new analytical method based on GC-QToF-MS is proposed for the enhanced characterisation and identification of mastic (*Pistacia* sp.) resin in archaeological samples. New insights into the use of mastic in ancient Egypt are provided.

## Graphical abstract

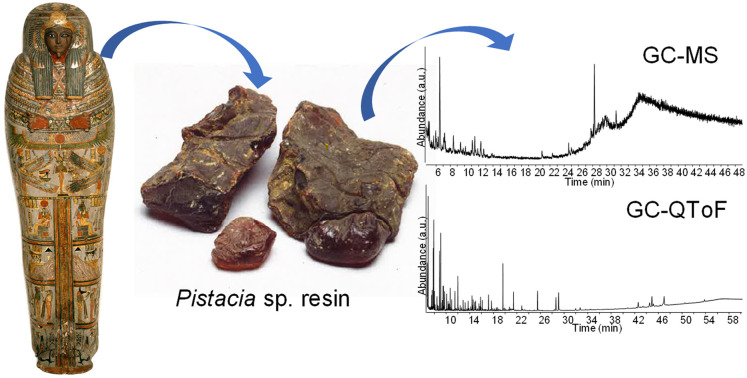

## Introduction

Mastic resin has been obtained for millennia from trees of the *Pistacia* genus (*Anacardiaceae* family) by collecting the exudate produced when the bark is cut or damaged. Four main *Pistacia* species are present in the Mediterranean area: *P. atlantica*, *P. khinjuk*, *P. lentiscus* and *P. terebinthus*. Today, only the cultivated *P. lentiscus* is commercially used to produce large quantities of resin. However, historically, the major source of mastic was the sub-species *P. lentiscus* L. *var. chia* from the Greek island of Chios.^1^

Mastic has been used as a varnish since ancient times,^2–5^ as well as many other applications, such as incenses,^6,7^ balsams,^8^ embalming materials,^9,10^ adhesives^11,12^ and coatings.^13^ Today, mastic is used mainly as chewing gum, but also for medicine, cosmetics, additives to food and as a sealant or filler in construction.^3^

**Fig. 1:**
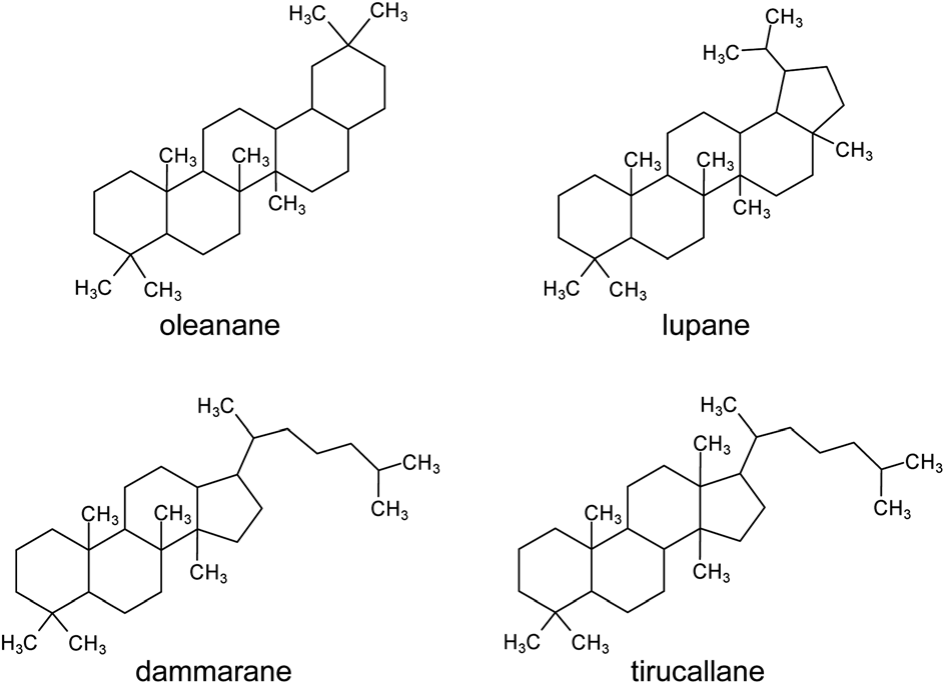
Molecular skeletons of the main triterpenoid classes found in *Pistacia* resin.

The chemical composition of the resin has been studied and found to contain a polymeric fraction (*cis*-1,4-poly-β-myrcene),^14^ a small fraction of mono- and sesquiterpenes, such as α- and β-pinene,^15,16^ and a main fraction composed of triterpenoids.^4,5,17–23^ The triterpenoids present in mastic resin have mostly tetra- and pentacyclic skeletons classified as oleananes, tirucallenes, dammaranes and lupanes (Fig. 1).^17,24^ Some bicyclic and tricyclic triterpenoids are also reported as minor components.^5,21^

The characterisation of triterpenoids is intrinsically complex. The compounds often share similar skeletons, sometimes differing only in the position of a double bond, and there are many isomers. Natural ageing pathways lead to formation of many derivative products that have not been fully characterised. However, mass spectrometry has demonstrated potential to differentiate between these molecules, as summarised by Assimopoulou and Papageorgiou.^17^ Several mass spectrometric techniques have been applied to the study of mastic, such as direct mass spectrometry (DTMS, DEMS and DIMS),^2,11,22,25,26^ matrix-assisted laser desorption/ionisation time-of-flight (MALDI-ToF),^23^ and laser desorption ionisation mass spectrometry (LDI-MS).^27^ However, the chromatographic separation of the triterpenoids is highly advantageous for molecular characterisation, as shown in numerous GC-MS studies.^4,5,7,12,20,21,28,29^ Analytical pyrolysis coupled to GC-MS (Py-GC-MS) has also been used^30–32^ and a few applications of high pressure liquid chromatography mass spectrometry (HPLC-MS) are reported.^19,30^ These studies have provided a good understanding of the molecular composition of mastic, but some discrepancies appear. In addition, further complications occur when degradation is considered. Although ageing products of mastic have been studied,^2,4–7,22,23,25–29,33^ the identification of mastic in historical and archaeological samples is usually based on the detection of a few markers, such as moronic, oleanonic, masticadienonic and isomasticadienonic acids, or the 28-norolean-17-en-3-one degradation product.^6,8–13,34,35^ The formation of the 28-norolean-17-en-3-one has been linked to exposure of the resin to heat^6^ and light,^4^ although it is not always detected, even in samples where resin degradation is evident and exposure to heat is highly suspected.^13^

Determination and interpretation of the composition of ancient mastic therefore remains a challenge not only due to the intrinsic complexity of triterpenoid constituents, but also to other materials commonly added to ancient formulations. High resolution and high accuracy mass spectrometry techniques are promising approaches to address these challenges, as the detailed information obtainable and the various operating modalities of these instruments enable further insights into molecular structures, enhanced isomer discrimination and additional tools for distinguishing molecules even when chromatographic separation is difficult.^30,36^

This study was undertaken with the aim to present a more complete characterisation of archaeological *Pistacia* resin by using gas chromatography coupled to quadrupole time-of-flight mass spectrometry (GC-QToF-MS). This represents the first application of the technique to the study of this material. Following a chromatographic optimisation, reference samples were investigated in different mass acquisition modalities, including low energy ionisation and tandem mass spectrometry to clarify the mass spectral features of the triterpenoid components. An additional goal was to test whether experiments could help us understand chemical changes induced by anthropogenic heating with particular attention to the formation of 28-norolean-17-en-3-one and whether this can always be considered a molecular marker for the heating of mastic.^6^ The experimental results were applied to interpret results obtained from archaeological samples containing mastic in different formulations, such as lumps of relatively pure resin, residues on ceramics, and varnish/coating layers. This part of the investigation focussed on exploring how chemical profiles of *Pistacia* resin present themselves in real archaeological samples from different degrees of preservation and use contexts, what the molecular profiles reveal about the resin properties from a use perspective, and whether such profiles correlate with the colour range of the material. The study also sought to address the question of how *Pistacia* resin was applied as varnish or coating. Being a solid at ambient temperature, the material must be transformed to a liquid or pseudo-liquid state to be applied as a thin layer. For example, when mastic varnish is applied to paintings, it is either in an “oil varnish” formulation obtained by boiling the natural resin in drying oils, such as linseed or walnut oil, or in a “spirit varnish” formulation (introduced in the 16th century) obtained by dissolving the natural resin in a volatile solvent, such as turpentine, which then mostly evaporates.^4^ Similar technologies might have been available in ancient Egypt, but no evidence of distillation – necessary to extract essential oils and fragrances – from Ancient Egypt has been reported so far.^37^ However, evidence for distillation exists in other regions, *e.g.* Mesopotamia, Slovakia, Sardinia, and Cyprus, from the end of the 5th millennium BCE to the 2nd millennium BCE.^38^

This study therefore represents a first step to address these complex questions starting from high-quality chemical data acquired by GC-QToF-MS.

## Experimental

### Samples

A reference sample of mastic resin from *P. lentiscus* L. *var. chia* was used for optimisation of the analytical procedure and the heating experiment. The sample was taken from material (provenance unknown) in the British Museum (London, UK) reference collection (REFC66498).

Archaeological samples from various objects were investigated. The samples are representative of different use and preservation contexts for the resin with potential exposure to different environmental conditions (marine waterlogged, heat, dry environment, *etc.*) resulting in different preservation states. They are varied in appearance, exhibiting colours in a range from yellow to orange to black.

Samples from the Uluburun shipwreck (Turkey, 1320 ± 15 BCE^39^) were collected as lumps of resin found inside ceramic transport containers known as Canaanite jars (Fig. S1 – ESI†). It is estimated that the ship carried over half a tonne of resin.^40,41^ GC-MS analysis confirmed the identity of this resinous material as *Pistacia* resin,^42^ but there has been some debate as to whether these jars contained resin only, or a mixture of resin and another product such as wine.^43^ However, no molecular evidence for wine biomarkers has been obtained so far.^44^

Samples of amorphous deposits were collected from ceramics from the 18th Dynasty (1548-1302 BCE) Pharaonic town on Sai Island in Upper Nubia (Sudan). The containers are blackened in appearance, suggesting their likely use as incense burners (Fig. S2, ESI†). Previous investigations reported the identification of *Pistacia* resin in these samples.^45^

Samples of varnishes and coatings on Egyptian coffins and cartonnage (plaster and linen) mummy cases from the collections of the British Museum (22nd Dynasty – 943-716 BCE) and Fitzwilliam Museum (range of dates from 18th Dynasty to Ptolemaic or Roman period, including some of unknown date) were also included. These were originally collected and studied in the framework of two different projects.^13,46^ The samples from the British Museum collection are all from surface coatings and include yellow and black varnish layers. The provenance of the objects is uncertain, and all were purchased in the 19th century, possibly in Thebes (Fig. S3 and S4, ESI†). The samples from the Fitzwilliam Museum collection were collected from yellow or black varnishes or coatings on coffins or cartonnage (or fragments thereof), in addition to some golden resin lumps (Fig. S5–S10, ESI†).

All the samples investigated are summarised in Table 1. As the analyses of these samples were performed over several years of research, it was not possible to re-analyse all of them with the optimised GC-QToF-MS method. This was due to a lack of residual sample and to the fact that most objects could not be re-sampled. Nevertheless, all analyses are included in the article, as they remain instrumental to discuss the use of *Pistacia* resin in different contexts.

**Table 1:** Description of the archaeological samples under investigation

Object registration number	Date	Sample name	Description	Analytical method
Uluburun, Canaanite jar KW 39	*ca.* 1320 BCE	KW 39	Yellow lump of resin found inside Canaanite jar number KW 39 from the Uluburun shipwreck, Turkey	C
Uluburun, Canaanite jar KW 49	*ca.* 1320 BCE	KW 49	Orange lump of resin found inside Canaanite jar number KW 49 from the Uluburun shipwreck, Turkey	C
Uluburun, Canaanite jar KW 144	*ca.* 1320 BCE	KW 144	Dark orange lump of resin found inside Canaanite jar number KW 144 from the Uluburun shipwreck, Turkey	C
Uluburun, Canaanite jar KW 215	*ca.* 1320 BCE	KW 215	Yellow lump of resin found inside Canaanite jar number KW 215 from the Uluburun shipwreck, Turkey	C
Uluburun, Canaanite jar KW 605	*ca.* 1320 BCE	KW 605	Orange lump of resin found inside Canaanite jar number KW 605 from the Uluburun shipwreck, Turkey	C
EA6666	22nd Dynasty (943-716 BCE)	C2_R1	Yellow varnish from below right hand on lid of ancient Egyptian coffin of Horaawesheb	B
EA29578	22nd Dynasty (943-716 BCE)	C3_R1	Yellow varnish from the collar of ancient Egyptian coffin of Padihorpakhered	B
		C3_R2	Yellow varnish from right side of head of ancient Egyptian coffin of Padihorpakhered	B and C
EA29577	22nd Dynasty (943-716 BCE)	C4_R2	Yellow varnish from left shoulder of ancient Egyptian mummy case of Djedameniuefankh	B
EA6685	22nd Dynasty (943-716 BCE)	C11_R1	Yellow varnish from below left side of hair of ancient Egyptian mummy case of Penpy	B and C
		C11_R2	Yellow varnish from left side of face of ancient Egyptian mummy case of Penpy	B
		C11_R3	Yellow varnish from right shoulder of ancient Egyptian mummy case of Penpy	B and C
EA6660	22nd Dynasty (943-716 BCE)	C13_R7	Yellow varnish from under right hand lappet of ancient Egyptian coffin of Denytenamun	B and C
EA9864	22nd Dynasty (943-716 BCE)	B8_R1	Black varnish from right side of foot of ancient Egyptian Osiris statue	B
		B8_R2	Black varnish from back of ancient Egyptian Osiris statue	B
E.64.1896	22nd Dynasty (943-716 BCE)	F01A	Varnish on surface of Nakhtefmut cartonnage	A
E.1.1822	21st Dynasty (1077-943 BCE)	F09A	Yellow resin from various areas of Nespawershefyt coffin (lid and base from both outer and inner coffin) and mummy board	A
		F09B		
		F09C		
		F09D		
		F09E		
		F09F		
		F09G		
		F09H		
		F09K		
E.GA.507.1947	21st Dynasty (1077-943 BCE)	F15A	Yellow varnish from coffin (face)	A
E.GA.528.1947	New Kingdom (1548-1069 BCE)	F16A	Yellow varnish from the surface of a coffin fragment	A
E.GA.504.1947	New Kingdom (1548-1069 BCE)	F17A	Black resin from coffin (face)	A
E.GA.2672.1943	Late 20th Dynasty (1189-1077 BCE)	F18A	Yellow varnish from a coffin fragment	A
E.558.1939	Unknown	F20A	Yellow varnish from wooden ear (fragment)	A
E.GA.6548.1943	22nd Dynasty (943-716 BCE)	F21A	Yellow varnish from cartonnage fragment	A
E.GA.2888.1943	22nd Dynasty (943-716 BCE)	F23A	Yellow varnish from cartonnage fragment (below wing)	A
E.GA.5851.1943	21st Dynasty (1077-943 BCE)	F26A	Black resin from a wooden lotus (coffin fragment)	A
E.GA.2891.1943	22nd Dynasty (943-716 BCE)	F27A	Golden resin from front of cartonnage fragment	A
E.GA.1174.1947	Late 20th Dynasty (1189-1077 BCE)	F28A	Golden resin from cartonnage fragment (chin)	A
E.GA.2870.1943	19th–20th Dynasty (1292-1077 BCE)	F30A	Yellow varnish from hand with Djed pillar	A
EGA.503.1947	Possibly New Kingdom (1548-1069 BCE)	F37A	Black resin from coffin face	A
E.200.1939	Late New Kingdom – Third Intermediate period	F43A	Black resin from coffin face (wig)	A
E.114.1903	18th Dynasty (1548-1302 BCE)	F45A	Detached fragment of amber-coloured resin from Abydos	A
E.133.1891	Ptolemaic or Roman period (332 BCE-641 CE)	F46A	Black resin from cartonnage fragment	A
E.W.94	26th Dynasty (664–525 BCE)	F52B	Reddish resin from ‘loop’ of resin on back leg of jackal figure	A
SAV1W 012/2017	18th Dynasty (1548-1302 BCE)	Sai 012	Black resin from a fragment of incense burner from Sai Island	B
SAV1W 0245/2015	18th Dynasty (1548-1302 BCE)	Sai 0245	Brown resin from a fragment of incense burner from Sai Island	B

### Heating experiment

A few mg of resin from the reference sample were placed on aluminium foil and then heated on a hot plate. Mild and intense heating conditions were applied, and different heating times were considered. Thus, the conditions adopted were:

• 100 °C for 4 hours

• 100 °C for 8 hours

• 250 °C for 30 minutes

• 250 °C for 2.5 hours.

Another experiment was conducted using an oven, in which the reference sample was heated up to 400 °C. The temperature was maintained for 10 min and then the oven was allowed to cool down. The entire process took approximately 3 hours. The analyses of these samples were performed in triplicate.

### Sample preparation and GC-MS conditions

The samples (approximately 0.1 mg) were powdered and solubilised using 500 μL of dichloromethane (DCM), which was then evaporated under nitrogen. The residue was derivatised using 100 μL of *N*,*O*-bis(trimethylsilyl)trifluoroacetamide (BSTFA) with 1% trimethylchlorosilane (TMCS) by heating the solution for 30 min at 70 °C. Three GC-MS systems and methods were used. Table 1 includes the analytical method(s) used to analyse each sample.

(A) A 6890 Agilent Technologies gas chromatograph coupled to a 5973 Agilent Technologies Mass Selective Detector single quadrupole mass spectrometer. The system was equipped with a split/splitless injector operated in spitless mode and kept at 250 °C. Column: HP-5MS column (30 m × 250 μm × 0.25 μm; Agilent Technologies, Cheadle, Cheshire, UK). Temperature programme: 35 °C, held for 1 min raised by 10 °C min^−1^ up to 325 °C, and held for 15 min. Carrier gas: helium (1 mL min^−1^). The MS transfer line temperature was kept at 280 °C. The MS ion source temperature was kept at 230 °C and the MS quadrupole temperature at 150 °C. The ionisation energy of the mass spectrometer was 70 eV (EI) and spectra were obtained in scanning mode between *m*/*z* 50 and 700.

(B) A 7890B Agilent Technologies gas chromatograph coupled to a 5977B Agilent Technologies single quadrupole mass spectrometer. A split/splitless injector (used in splitless mode) was maintained at 300 °C and a HP-5MS column (30 m × 250 μm x 0.25 μm; Agilent Technologies, Cheadle, Cheshire, UK) was used. Helium was used as carrier gas (1 mL min^−1^). The oven temperature was set at 40 °C, raised by 10 °C min^−1^ up to 200 °C, then raised by 3 °C min^−1^ until 325 °C, and held for 5 min. The MS transfer line temperature was kept at 280 °C. The MS ion source temperature was kept at 230 °C and the MS quadrupole temperature at 150 °C. The ionisation energy of the mass spectrometer was 70 eV and spectra were obtained in scanning mode between *m*/*z* 50 and 800.

(C) A 7250 Agilent Technologies GC-QToF system. A split/splitless injector (used in splitless mode) was maintained at 300 °C and a HP-5MS column (30 m × 250 μm × 0.25 μm; Agilent Technologies, Cheadle, Cheshire, UK) was used. Helium was used as carrier gas (1 mL min^−1^). The oven temperature programme was optimised (see Results section). The final conditions adopted were: initial temperature 40 °C, then raised by 10 °C min^−1^ up to 250 °C, then raised 2 °C min^−1^ up to 320 °C, and held for 5 min. The MS transfer line temperature was kept at 300 °C. The MS ion source temperature was kept at 200 °C and the MS quadrupole temperature at 150 °C. The spectral range was between 50 and 700 *m*/*z* with an acquisition rate of 5 spectra per s. Standard energy ionisation (EI) and low energy ionisation (low EI) experiments were performed. In standard EI, the electron energy was 70 eV and the emission current was 4.0 μA. In low EI, the electron energy was 15 eV and the emission current was 0.5 μA. Targeted MS/MS experiments were also performed in low EI mode with acquisition time 200 ms per spectrum and using He as collision gas with collision energy 35 eV.

## Results and discussion

### Optimisation of analytical conditions for GC-QToF-MS (method C)

**Fig. 2:**
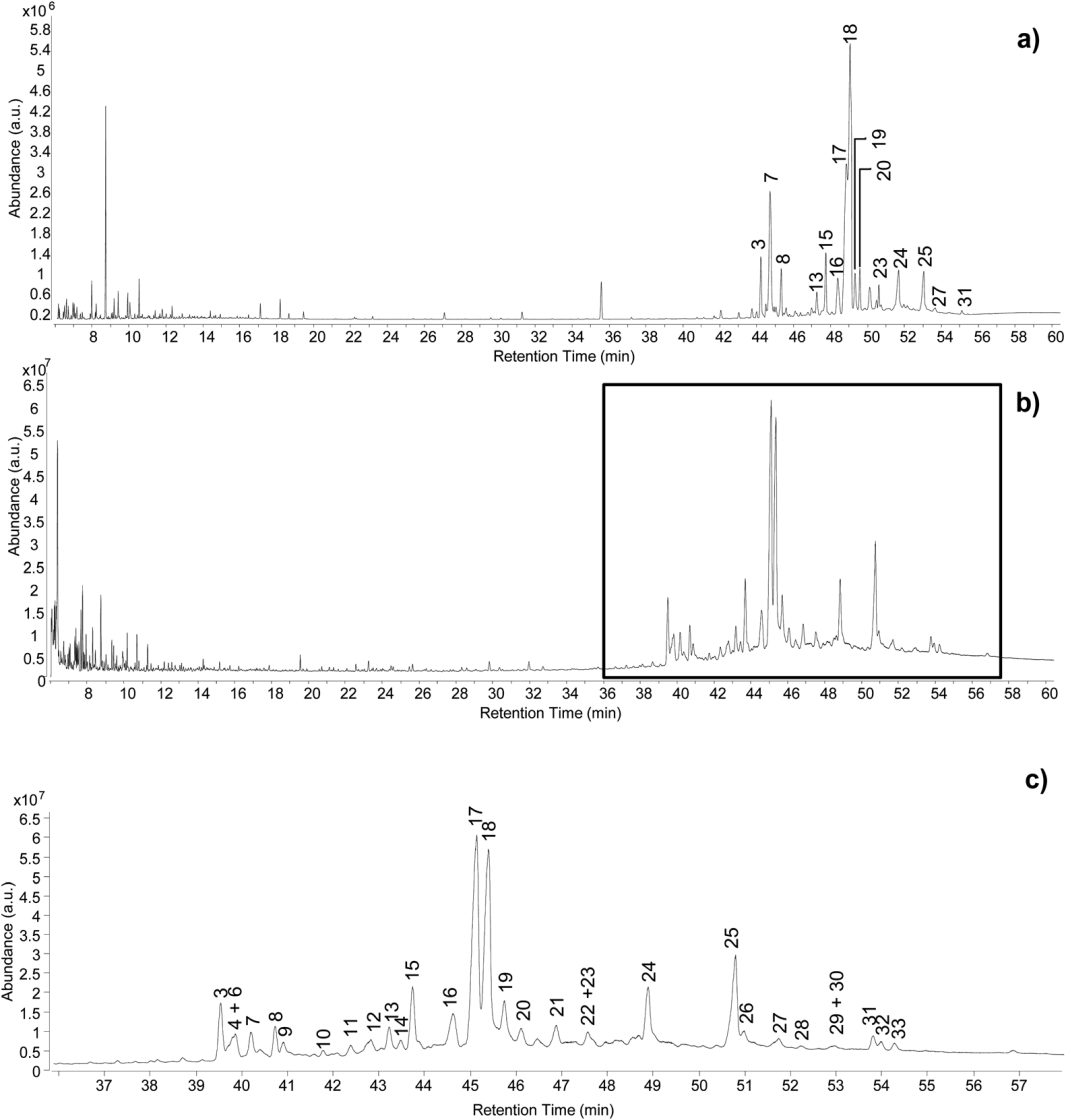
Chromatographic profiles obtained from the analysis of the reference sample by (a) GC-MS single quadrupole, (b) GC-QToF-MS (optimised conditions – Experimental section), (c) expanded view of the chromatographic region indicated in (b) between 36 and 58 min. Labels refer to Table 2. © The Trustees of the British Museum. Shared under a Creative Commons Attribution-NonCommercial-ShareAlike 4.0 International (CC BY-NC-SA 4.0) licence.

The optimisation of the chromatographic conditions was performed on the reference sample (REFC66498) and started with parameters previously optimised on method B. Chromatograms obtained using these conditions already showed a good quality of peak separation. However, coelution of some peaks was evident in the part of the chromatogram containing the triterpenoid fraction (Fig. 2a). Method B was initially translated onto the 7250 Agilent Technologies GC-QToF-MS system, and then the temperature programme was progressively modified with the intention to accelerate the first part of the run (up to 250 °C) and to slow down the second part of the run (up to 320 °C), which corresponded to the elution of most triterpenoids. The best result was obtained with the conditions reported in the Experimental section, producing the chromatogram shown in Fig. 2b. Although complete separation of certain peaks was not fully achieved, a significant improvement was obtained, and the triterpenoid fraction eluted over a wider time range. Further deceleration of the run from 2 °C min^−1^ to 1 °C min^−1^ between 250 °C and 320 °C was tested but did not produce any significant improvement and resulted in a time of analysis that was considered unacceptably long (from 61 to 96 min). It must be considered that the chemical structures of some of these triterpenoids are very similar, thus their complete chromatographic separation is virtually impossible in a monodimensional system and in an acceptable analytical time suitable for routine analysis.

The inlet parameters were tested as well. 1 : 5 and 1 : 10 split ratios resulted in a significant decrease in sensitivity and increase in the signal-to-noise ratio (Fig. S11, ESI†). Considering that the method was optimised with the aim of applying it to small archaeological samples, splitless conditions appeared as the most suitable to obtain the highest sensitivity.

**Fig. 3:**
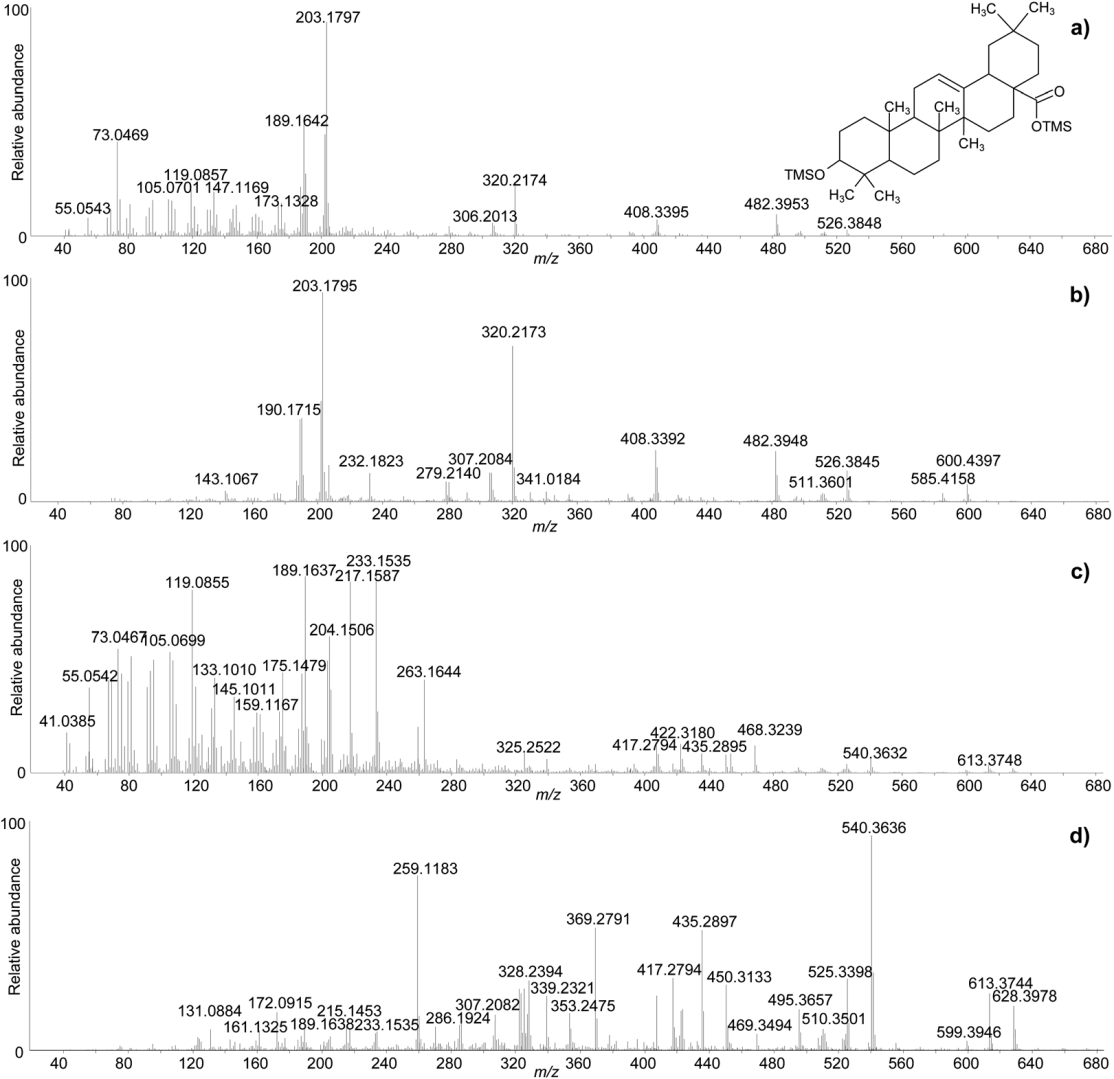
Comparison between the accurate mass spectra of oleanolic acid (2TMS) obtained with standard EI (a) and low EI (b), and ‘unknown 628’ obtained with standard EI (c) and low EI (d). © The Trustees of the British Museum. Shared under a Creative Commons Attribution-NonCommercial-ShareAlike 4.0 International (CC BY-NC-SA 4.0) licence.

One of the difficulties in the identification of triterpenoids by GC-MS is that the molecular ions, from which the mass of the molecules can be inferred, are often not obvious in the mass spectra. This is particularly frequent for derivatised molecules, and even more so for trimethylsilylated derivatives, as the mass of the non-derivatised molecule is significantly increased by the relatively large trimethylsilyl groups. Moreover, in the EI fragmentation of trimethylsilylated molecules with multiple derivatisation sites, [M-15]^+^ and [M-31]^+^ ions tend to be more abundant than the molecular ions, which are sometimes not detectable at all.^47^ For the purpose of structural elucidation, soft ionisation experiments, *e.g.* chemical ionisation, are useful in these cases, in order to maximise the yield of the molecular ions.^2,26^ The GC-QToF-MS system used in this study enables low energy ionisation (low EI) mode to be used (see Experimental section). Hence, the potential of low EI was tested to clarify the mass of some derivatised triterpenoids. Fig. 3 shows a comparison between mass spectra of the same molecules (oleanolic acid and ‘unknown 628’) obtained with standard EI and low EI. The enhancement of the relative abundance of the molecular ions and other high-mass ions is evident in low EI conditions. However, this is counterbalanced by the absence or relative decrease of the lower-mass fragment ions, as a result of reduced fragmentation. As the low EI conditions also include a reduced emission current compared to standard EI (see Experimental section), the overall sensitivity of the low EI analytical mode is lower than standard EI, as shown in Fig. S12 (ESI).† As a result, low EI mode proved fundamental to clarify the mass of certain molecules and should be used as a refining analytical tool. However, attention must be paid to the fact that compounds present at low concentration might not ionise enough to be detected in low EI mode.

**Fig. 4:**
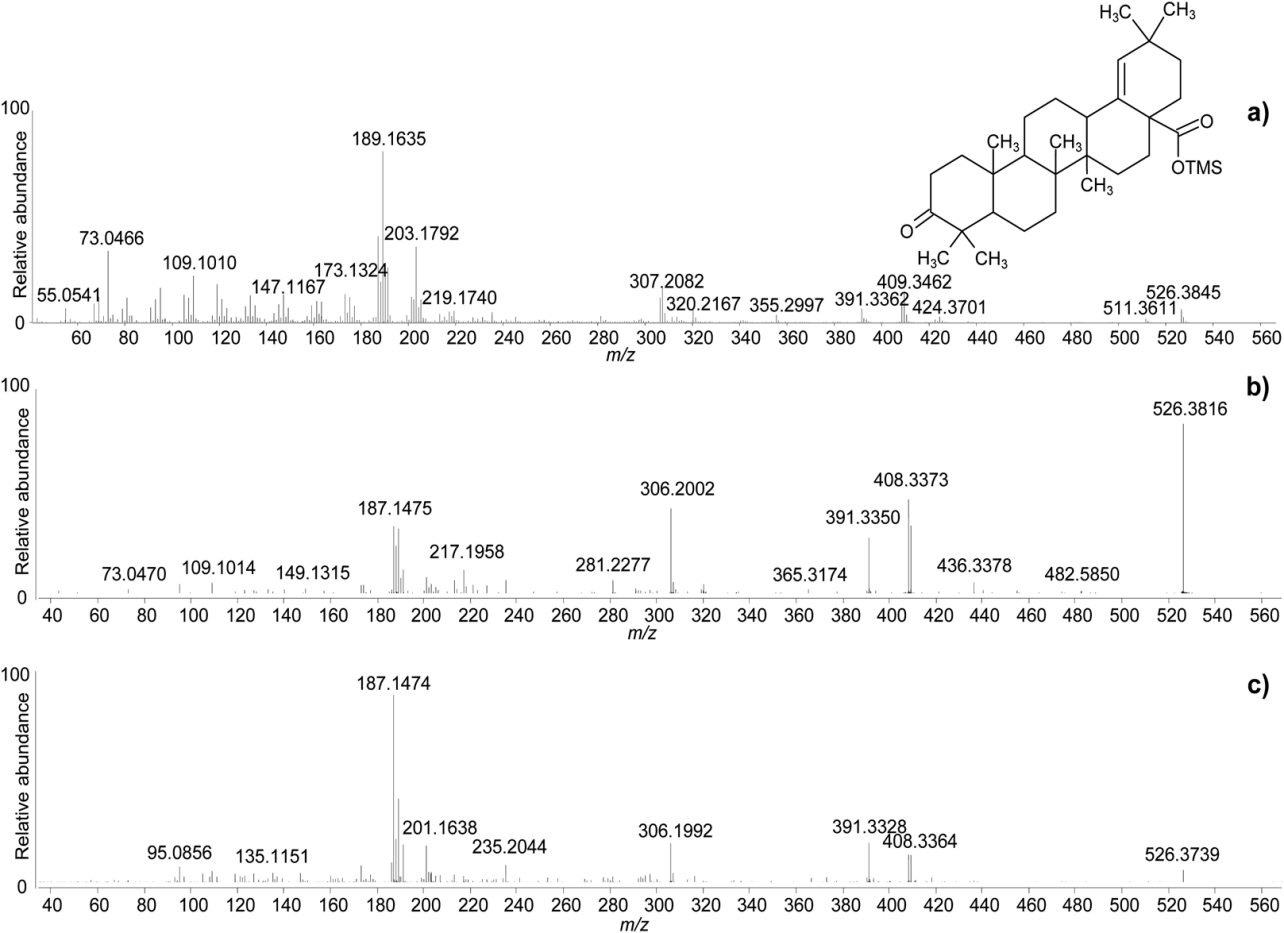
Accurate mass spectra of moronic acid (TMS) obtained with standard EI (a), and with MS/MS of the molecular ion with CID 25 V (b), and CID 40 V (c). © The Trustees of the British Museum. Shared under a Creative Commons Attribution-NonCommercial-ShareAlike 4.0 International (CC BY-NC-SA 4.0) licence.

The clarifications obtained on the molecular ions and the accurate mass values provided by the QToF analyser enabled raw chemical formulas to be assigned to most components (Table 2) with a difference between the experimental and calculated *m*/*z* values (dppm) below 2 ppm in all cases. The next step was the assignment of molecular structures. The available literature on this topic was taken as a starting point, as fragmentation pathways have been studied and some standard molecules analysed.^4,17,19–21,26,28,29^ However, the available data on trimethylsilylated triterpenoids are limited.^4,7,10,13,29^ Hence, the high-resolution mass spectra were interpreted exploiting the high mass accuracy of the various molecular fragments. Additionally, MS/MS experiments were performed to further understand the potential of GC-QToF-MS analysis in this type of application. As the mechanisms of electron impact (EI) fragmentation and collision-induced dissociation (CID) can be different, some molecular ions were fragmented in MS/MS mode by using different collision energies between 20 and 40 V. The results showed that, despite some differences in the relative abundances of the fragment ions, the main fragmentation pathways remain the same in the two modalities (Fig. 4). Nevertheless, MS/MS mode permits isolation of any fragment ion present in the EI mass spectra followed by selected fragmentation. This enables further structural elucidation power to be exploited.

**Table 2:** List of triterpenoid compounds detected by GC-QToF-MS in the reference sample of *Pistacia* resin and MS details. Numbers are used in peak labels. Some of the identifications remain tentative.

No	Compound	Retention time	Raw formula	Calculated *m/z*	Experimental *m/z*	dppm	Fragment ions (standard EI)*
1	**22,23,24,25,26,27-hexakisnor-dammaran-3,20-dione**	31.95	C_24_H_38_O_2_	358.2872	358.2875	0.89	340.2795, **315.2689**, 297.2581, 273.2215, 219.1745, 205.1590, 189.1639, 177.1272, 163.1482, 147.1170, 135.1169, 121.1012, 109.1012, 95.0856, 81.0699, 67.0542, 55.0543
2	**22,23,24,25,26,27-hexakisnor-dammaran-3-hydroxy-20-one (TMS)**	32.66	C_27_H_48_O_2_Si	432.3423	432.3424	-0.13	417.3188, 389.3236, 375.3081, 342.2919, 327.2685, **303.2678**, 302.2605, 299.2734, 285.2578, 191.1791, 189.1637, 175.1481, 161.1324, 143.0884, 135.1167, 121.1011, 109.1010, 95.0854, 81.0698, 73.0467, 69.0698, 55.0542
3	**Nor-β-amyrone**	39.49	C_29_H_46_O	410.3554	410.3549	1.3	395.3291, **204.1840**, 189.1637, 175.1480, 161.1323, 148.1233, 133.1011, 119.0588, 105.0698, 95.0854, 81.0698, 69.0698, 55.0542
	28-Norolean-12-en-3-one						
4	**Tirucallol (TMS)**	39.81	C_33_H_58_OSi	498.4257	498.4263	1.22	483.4032, 393.3529, 241.1954, **218.2034**, 203.1798, 189.1641, 175.1483, 161.1327, 147.1170, 133.1014, 119.0857, 109.1014, 95.0857, 81.0700, 73.0469, 69.0700, 55.0543
	Tirucalladienol						
5	**28-Norolean-17-en-3-one**	40.15	C_29_H_46_O	410.3550	410.3549	0.33	395.3304, 191.1790, 175.1477, **163.1479**, 147.1160, 133.1009, 119.0852, 105.0697, 95.0853, 81.0697, 69.0696, 55.0541
6	**β-Amyrone**	40.19	C_30_H_48_O	424.3705	424.3701	-0.98	406.3231, 393.3517, 383.2945, 365.2838, **218.2030**, 203.195, 189.1638, 175.1480, 161.1324, 147.1166, 133.1010, 119.0854, 107.0854, 95.0854, 81.0698, 69.0697, 55.0541
	Olean-12-en-3-one						
7	**Unknown_498_1**	40.21	C_33_H_58_OSi	498.4257	498.4262	1.02	483.4023, 408.3390, 393.3519, 339.3047, 279.2141, 229.1952, 218.2030, 204.1872, **189.1638**, 175.1483, 161.1325, 147.1168, 135.1166, 119.0855, 109.1011, 95.0855, 81.0698, 69.0698, 55.0542
	isomer of β-amyrin						
8	**β-amyrin**	40.68	C_33_H_58_OSi	498.4257	498.4259	0.42	393.3162, **218.2033**, 203.1798, 189.1641, 173.1328, 161.1327, 147.1170, 133.1013, 119.0857, 105.0700, 95.0856, 81.0700, 69.0699, 55.0543
	Olean-12-en-3-ol						
9	**Olean-18-en-3-ol (TMS)**	40.86	C_33_H_58_OSi	498.4257	498.4260	0.62	483.4019, 408.3389, **393.3518**, 385.2923, 339.3046, 279.2135, 255.2105, 241.1949, 229.1949, 218.2027, 203.1793, 189.1637, 175.1480, 161.1323, 147.1167, 135.1166, 121.1010, 109.1010, 93.0697, 81.0697, 73.0467, 69.0697, 55.0541
10	**Unknown_514 (TMS)**	41.73	C_32_H_54_O_3_Si	514.3842	514.3837	-1.01	496.3729, 481.3496, 420.3392, 406.3228, 392.3063, 379.2995, 283.2055, 253.1948, 239.1792, 218.2026, 204.1867, **189.1637**, 175.1481, 161.1324, 145.1012, 133.1011, 121.1011, 107.0855, 95.0855, 81.0698, 69.0698, 55.0542
11	**3,4-Seco-28-nor-olean-12-en-3,28-dioic acid (2TMS)**	42.30	C_36_H_64_O_4_Si_2_	616.4343	616.4348	0.79	601.4105, 526.3837, 499.3958, 409.3453, 391.3358, 320.2164, 309.2235, 202.1715, **189.1638**, 175.1480, 161.1322, 143.1061, 133.1010, 119.0854, 107.0854, 98.0854, 81.0698, 73.0468, 69.0698, 55.0542
12	**3,4-Seco-28-nor-olean-18-en-3,28-dioic acid (2TMS)**	42.78	C_36_H_64_O_4_Si_2_	616.4343	616.4345	0.3	601.4054, 526.3844, 498.3848, 409.3451, 320.2166, **203.1792**, 189.1637, 173.1324, 145.1011, 119.0855, 107.0854, 98.0854, 81.0698, 73.0468, 69.0698, 55.0542
13	**Unknown 408**	43.18	C_29_H_44_O	408.3392	408.3396	0.94	393.3153, 215.1790, **202.1712**, 189.1637, 173.1324, 159.1167, 145.1011, 131.0861, 119.0855, 105.0698, 91.0542, 81.0698, 69.0698, 55.0542
	likely a norolean-dienone						
14	**Unknown 524_1 (TMS)**	43.43	C_33_H_52_O_3_Si	524.3686	524.3688	0.43	509.3439, **406.0840**, 391.3361, 239.1792, 187.1481, 143.0867, 73.0467
15	**Oleandienone**	43.69	C_30_H_46_O	422.3549	**422.3550**	0.32	407.3300, 393.3153, 216.1864, 203.1792, 188.1549, 175.1480, 161.1323, 145.1011, 131.0861, 119.0855, 105.0698, 95.0854, 81.0698, 69.0698, 55.0542
	uncertain position of the double bonds						
16	**20,24-epoxy-25-hydroxy-dammaran-3-one (TMS)**	44.59	C_30_H_50_O_3_	458.3758	458.3760	-0.43	443.2979, 407.3308, 399.0058, 381.3149, 205.1588, 175.1483, 161.1326, **143.0873**, 125.0962, 107.0856, 95.0856, 85.0648, 59.0312
17	**Moronic acid (TMS)**	45.09	C_33_H_54_O_3_Si	526.3842	526.3852	1.86	511.3611, 424.3701, 409.3462, 391.3362, 355.2997, 320.2167, 307.2082, 219.1740, 203.1792, **189.1635**, 173.1324, 161.1324, 147.1167, 133.1010, 119.0854, 109.1010, 95.0854, 81.0697, 73.0466, 69.0697, 55.0541
	3-oxoolean-18-en-28-oic acid						
18	**Oleanonic acid (TMS)**	45.36	C_33_H_54_O_3_Si	526.3842	526.3851	1.67	511.3607, 408.3387, 391.3361, 320.2166, 307.2081, **203.1792**, 189.1637, 173.1324, 133.1011, 119.0855, 105.0698, 95.0854, 81.0698, 73.0467, 55.0542
	3-oxoolean-12-en-28-oic acid						
19	**Betulonic acid (TMS)**	45.70	C_33_H_54_O_3_Si	526.3842	526.3849	1.29	511.3607, 444.3045, 409.3451, 354.2543, 307.2081, 226.1693, 203.1792, **189.1637**, 175.1480, 159.1167, 133.1011, 119.0855, 107.0855, 95.0854, 81.0698, 73.0467, 55.0542
	3-oxolup-20(29)-en-28-oic acid						
20	**Oleanolic acid (2TMS)**	46.06	C_36_H_64_O_3_Si_2_	600.4394	600.4390	-0.66	585.4163, 526.3848, 482.3953, 408.3395, 320.2174, 306.2013, **203.1797**, 189.1642, 173.1328, 159.1169, 147.1169, 133.1013, 119.0857, 105.0701, 95.0857, 81.0700, 73.0469, 55.0543
	3-hydroxyolean-12-en-28-oic acid						
21	**Unknown 612 (2TMS)**	46.84	C_36_H_60_O_4_Si_2_	612.4030	612.4033	0.47	597.3800, 571.3639, 524.3682, 511.3600, 494.3573, 479.3341, 441.3188, 427.3033, 409.3458, 351.2682, 309.2577, 203.1792, 189.1638, 161.1325, 131.0873, 119.0855, 107.0855, 95.0855, 81.0698, **73.0467**, 55.0542
22	**Olean-18-en-3,28-diol (2TMS)**	47.38	C_36_H_66_O_2_Si_2_	586.4601	586.4600	-0.23	571.4367, 496.4093, 481.3866, 391.3362, 309.2574, 295.2419, 281.2262, 255.2104, 241.1949, 229.1947, 215.1789, 203.1791, 189.1636, 173.1323, 157.1029, 143.0878, 135.1166, 121.1010, 107.0854, 95.0855, 81.0698, **73.0467**, 69.0698, 55.0542
23	**Unknown 614_1 (2TMS)**	47.52	C_36_H_62_O_4_Si_2_	614.4187	614.4181	-0.92	599.3967, 524.3680, 468.0987, 422.3546, 407.3300, 262.1919, 216.1864, **203.1792**, 189.1637, 143.0867, 133.1011, 119.0855, 107.0855, 95.0854, 81.0698, 73.0467, 55.0542
24	**Masticadienonic acid (TMS)**	48.80	C_33_H_54_O_3_Si	526.3842	526.3851	1.67	**511.3607**, 493.3490, 436.3334, 421.3102, 393.3153, 307.2081, 257.1898, 243.1740, 213.1635, 185.1324, 169.1018, 143.0867, 121.1011, 95.0854, 73.0467, 55.0542
	(*24Z*)-3-oxolanosta-7,24-dien-26-oic acid						
25	**Isomasticadienonic acid (TMS)**	50.68	C_33_H_54_O_3_Si	526.3842	526.3847	0.91	**511.3607**, 493.3490, 436.3334, 421.3102, 393.3153, 307.2081, 257.1898, 243.1740, 213.1635, 185.1324, 169.1018, 143.0867, 121.1011, 95.0854, 73.0467, 55.0542
	(*24Z*)-3-oxolanosta-8,24-dien-26-oic acid						
26	**Unknown 614_2 (2TMS)**	50.94	C_36_H_62_O_4_Si_2_	614.4187	614.4181	-0.92	**599.3967**, 526.3842, 511.3614, 493.3506, 476.3147, 461.2910, 421.3111, 393.3158, 305.1935, 257.1901, 187.1482, 169.0682, 159.1169, 145.1013, 133.1013, 119.0856, 105.0700, 95.0856, 73.0469, 55.0543
27	**3-O-acetyl-masticadienonic acid (TMS)**	51.70	C_35_H_58_O_4_Si	570.4104	570.4106	0.29	555.3869, **495.3641**, 423.3244, 295.2058, 227.1790, 213.1635, 187.1481, 159.1167, 147.0656, 135.0624, 119.0855, 107.0855, 95.0854, 81.0698, 73.0467, 55.0542
28	**Unknown 614_3 (2TMS)**	52.03	C_36_H_62_O_4_Si_2_	614.4187	614.4180	-1.08	599.3950, 540.3633, 509.3447, 469.3131, 419.2948, 391.2993, **325.2528**, 259.1182, 169.0679, 159.1168, 143.0871, 133.1012, 121.1011, 107.0855, 95.0855, 81.0699, 73.0468, 67.0542, 55.0542
29	**Unknown 526 (TMS)**	52.65	C_33_H_54_O_3_Si	526.3842	526.3835	-1.37	511.3613, 493.3496, 443.2972, **421.3109**, 403.2999, 393.3155, 365.2838, 311.2369, 257.1901, 187.1482, 173.1325, 159.1168, 145.1011, 133.1011, 119.0855, 105.0699, 95.0855, 81.0698, 73.0468, 67.0542, 55.0542
	isomer of masticadienonic acid						
30	**11-oxo-masticadienonic acid (TMS)**	52.75	C_33_H_52_O_4_Si	540.3629	540.3635	1.04	525.3401, 512.3674, 443.2983, 421.3102, 369.2739, 259.1691, **233.1537**, 187.1480, 169.0679, 145.1012, 131.0873, 119.0855, 107.0855, 95.0854, 81.0698, 73.0468, 67.0542, 55.0542
31	**3-O-acetyl-isomasticadienonic acid (TMS)**	53.77	C_35_H_58_O_4_Si	570.4104	570.4107	0.46	555.3869, **495.3641**, 423.3244, 295.2058, 227.1790, 213.1635, 187.1481, 159.1167, 147.0656, 135.0624, 119.0855, 107.0855, 95.0854, 81.0698, 73.0467, 55.0542
32	**Unknown 658 (2TMS)**	53.94	C_38_H_66_O_5_Si_2_	658.4449	658.4446	-0.42	643.4212, 538.3490, 511.3607, 495.3660, 468.3238, 453.3001, 423.3257, 407.2945, 263.1643, 233.1537, 217.1589, 204.1505, 189.1639, 175.1481, 161.1324, 147.1167, 133.1012, **119.0856**, 107.0856, 95.0855, 81.0699, 73.0469, 69.0699, 55.0542
33	**Unknown 628 (2TMS)**	54.21	C_36_H_60_O_5_Si_2_	628.3979	628.3981	0.27	613.3751, 599.3947, 540.3634, 525.3400, 495.3657, 450.3132, 435.2900, 417.2793, 407.2945, 369.2792, 339.2322, 325.2525, **259.1184**, 187.1482, 169.0680, 159.1167, 145.1012, 131.0867, 119.0855, 107.0856, 95.0855, 81.0699, 73.0469, 67.0542, 55.0543
	It contains the 11-oxo functionality						

By taking advantage of the various analytical modalities and data processing described so far, the composition of the reference sample of *Pistacia* resin was characterised and the results are summarised in Table 2. In addition to the triterpenoid fraction, few peaks attributable to monoterpenes were present in the first part of the chromatogram (Fig. 2b), together with a lipidic fraction represented by small peaks attributed to palmitic acid, stearic acid, monopalmitin and monostearin. The most common identified compounds were in agreement with the literature.^2,10,17,19,20,26,29^ However, the EI mass fragmentation rules described in the literature for these molecules do not take into account trimethylsilyl derivatives,^17^ which have some specific EI fragmentation rules.^47–49^ As a result, some mass spectral interpretations remained challenging in terms of assigning an unequivocal molecular structure to some detected compounds, although the assignment of raw formulas and characteristic fragment ions suggested the presence of specific skeletons and functionalities. Despite the incomplete disclosure of all molecular structures, this dataset represents the first database containing accurate mass details of trimethylsilyl derivatives of triterpenoids present in *Pistacia* resin and reports a higher number of hydroxylated compounds compared to the literature, likely due to the enhanced possibility of detecting high mass molecular ions by GC-QToF-MS.

### Heat-induced modifications

The samples showed visual differences during and at the end of the heating process. The resin began to melt slightly below 100 °C. A colour change started to occur after 4 hours of exposure at 100 °C (Fig. S13a, ESI†) and some orange areas in a yellow matrix were observed after 8 hours (Fig. S13b, ESI†). Upon exposure to 250 °C the resin immediately turned a deep orange/caramel colour, which kept darkening with time (Fig. S13c, ESI†). Exposure to 400 °C led to a black residue that was difficult to separate from the aluminium foil (Fig. S13d ESI†).

**Fig. 5:**
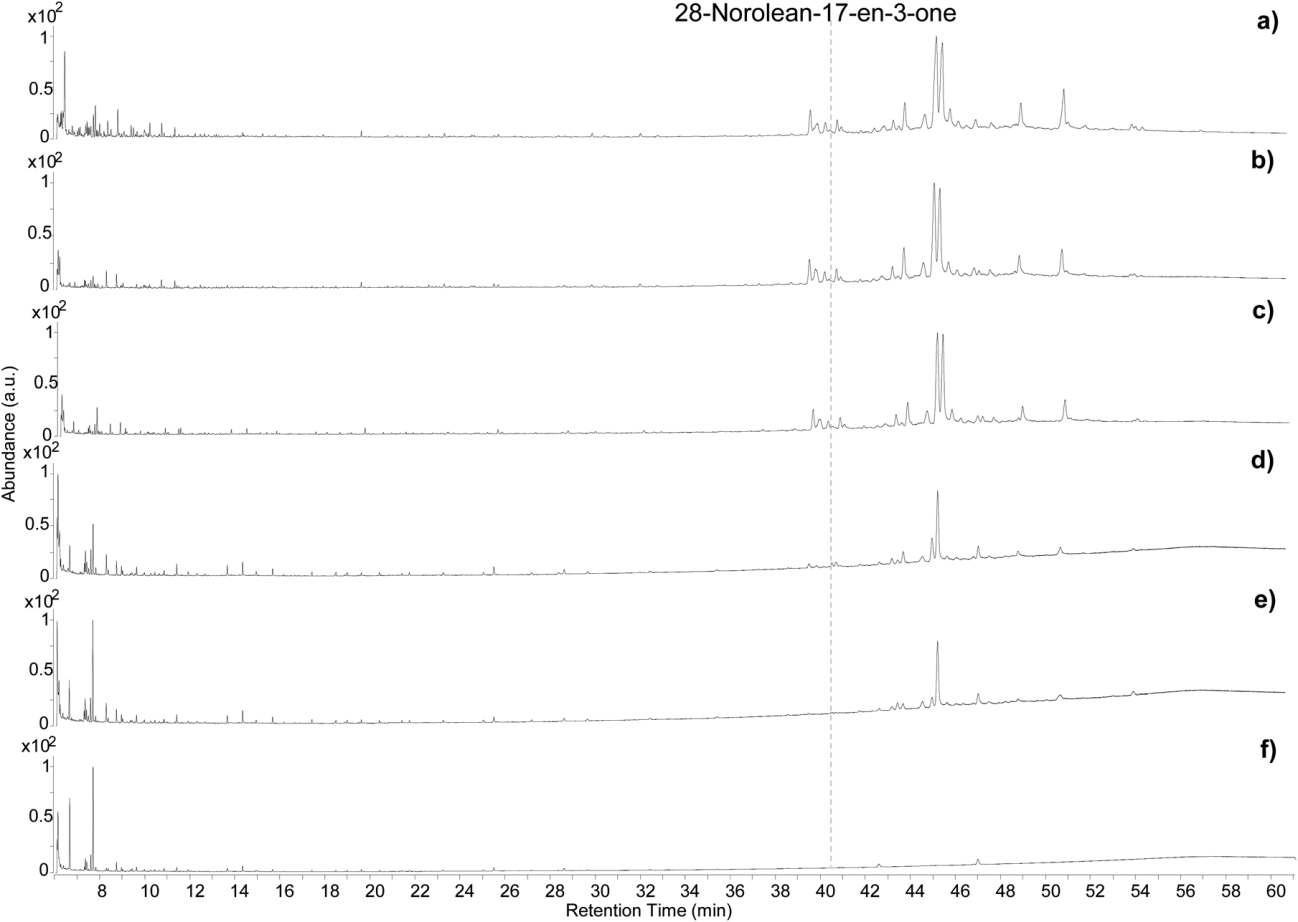
Chromatographic profiles obtained by GC-QToF analysis of the *Pistacia* reference sample (a), and the samples from the heating experiment at 100 °C for 4 hours (b), 100 °C for 8 hours (c), 250 °C for 0.5 hours (d), 250 °C for 2.5 hours (e), 400 °C for 10 min (f). © The Trustees of the British Museum. Shared under a Creative Commons Attribution-NonCommercial-ShareAlike 4.0 International (CC BY-NC-SA 4.0) licence.

The five samples were analysed by GC-QToF-MS and the chromatographic profiles are reported in Fig. 5. An expanded view of the normalised chromatograms is shown in Fig. S14 (ESI).† After exposure to 100 °C for 4 and 8 hours the main qualitative change was related to a slight progressive relative reduction in the abundance of masticadienonic acid and isomasticadienonic acids. By contrast, after 30 min of heating at 250 °C, the compositional changes were more evident. The smaller triterpenoids eluting around 40 min underwent a drastic reduction, and the relative abundance between moronic and oleanonic acid also changed. Oleanonic acid proved to be far more stable to heat than moronic acid. Furthermore, 28-norolean-17-en-3-one, not detected in the other chromatograms discussed so far, was formed in these conditions, and appeared with significant abundance. Extending the heating time at 250 °C for 2 additional hours led to a further general reduction of all compounds, with oleanonic acid remaining among the few compounds with significant relative abundance. Interestingly, 28-norolean-17-en-3-one also showed a reduction in its relative abundance. The heating experiment at 400 °C completely charred the resin and no residual markers were detected. The small peaks observed in the chromatogram (Fig. 5f) are not related to triterpenoids.

As this experiment was designed to study the possible formation of 28-norolean-17-en-3-one as a marker of heating in an open environment, we observed that this compound forms in the applied conditions when the resin is exposed to relatively high temperatures (above 100 °C). A relative increase in abundance appears to occur by increasing the temperature and extending the time of the heating. However, the compound is not stable to prolonged heating nor very high temperatures. This makes 28-norolean-17-en-3-one a reliable marker for heating of *Pistacia* resin when it is detected. However, its lack of detection in an archaeological sample does not imply that the resin was not heated, as 28-norolean-17-en-3-one might have further degraded in the case of high or prolonged heat. Nevertheless, the different stability to heat of moronic and oleanonic acids appears as another useful parameter. Samples showing a significantly higher relative abundance of oleanonic acid compared to moronic acid might be indicative of the resin having undergone heat treatment regardless of the presence of 28-norolean-17-en-3-one. These results add to previous observations on this subject.^4,6^

### Archaeological samples

#### Uluburun – resin lumps

**Fig. 6:**
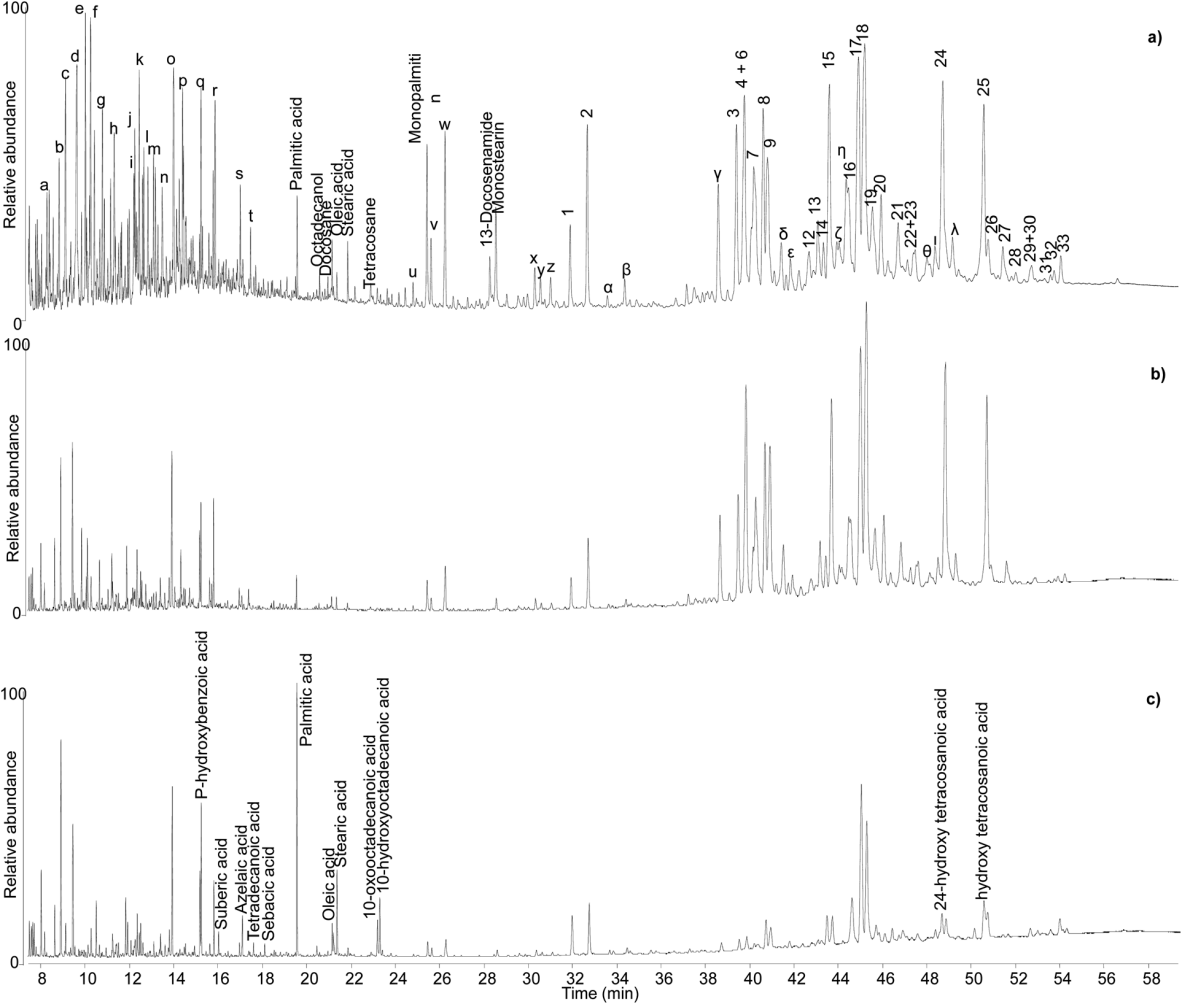
Chromatographic profiles obtained by GC-QToF analysis of samples KW39 (a), KW49 (b), and KW144 (c). Numbers refer to Table 2. Letters refer to Table S1.† © The Trustees of the British Museum. Shared under a Creative Commons Attribution-NonCommercial-ShareAlike 4.0 International (CC BY-NC-SA 4.0) licence.

The five samples from the Uluburun shipwreck generally showed an extremely good level of preservation. In particular, the profile obtained for sample KW 39 showed all the compounds described for the reference sample of *Pistacia* resin, in addition to several other triterpenoids and a high number of smaller molecules, mostly monoterpenes (Fig. 6a). The main compounds that were not present in the reference sample – and in Table 2 – are marked with letters and are listed in Table S1.†

Sample KW 49 showed a similar profile to sample KW 39 apart from a lower relative abundance of volatile molecules (Fig. 6b), probably due to a slightly less favourable preservation state. The chromatographic profiles obtained for samples KW 215 and KW 605 were extremely similar to that of sample KW 49. By contrast, sample KW 144 showed some differences compared to other samples. In addition to a general lower relative abundance of both triterpenoids and small molecules, aliphatic mono-, di-, and hydroxy/oxo carboxylic acids were also detected, which suggest the presence of a lipid fraction in this sample (Fig. 6c). In addition to being less well preserved, sample KW 144 appears to have been mixed with a lipid material that had undergone oxidation, as inferred by the relatively high abundance of dicarboxylic and hydroxy/oxo acids.^50^ The exact source of the lipid material is unclear, but it could have been the result of deliberate mixing of a plant oil with the resin to obtain a sort of “oil varnish” formulation that would help to get the resin into a liquid state necessary to fill the Canaanite jars (see discussion of varnishes below). However, an unintentional post-depositional mixing cannot be excluded. Stirrup jars thought to have contained olive oil were also found on the Uluburun shipwreck,^51^ and it remains a strong possibility that contents from a variety of jars spilled into the hull of the ship during the sinking event causing unintentional mixing on the seafloor. As evidence of oil markers was not obtained for the other Uluburun samples, the liquid or pseudo-liquid state might have been reached with a different method, which could involve heating. However, no heating markers were detected, but most importantly some of the jars contained clumps of detritus at their bases, which included *Pistacia* fruit and leaves, insects, snails, dirt, bark fragments, *etc.* Had the resin been heated and poured into the jars as a liquid, it seems likely that the detritus would have remained in the heating vessel or perhaps been filtered out with sieves during the filling. It appears more likely that the resin was filled as solid pellets or small chunks and droplets in the state they were collected from the trees, and then the jars were tightly sealed. Heating and melting phenomena could still have occurred, as the jars would have been heated under the sun, potentially melting the resin within, and the solid bits in the resin lumps would sink to the bottom of the vessel. The temperatures reached inside the jars sitting under the sun all day could be sufficiently high to melt the resin, as our heating experiments show that melting occurs at relatively low temperatures, but not high enough to significantly alter the composition of the resin, again in agreement with our observations.

The monoterpenoid fraction in these samples might, at least partially, be part of the resin composition. These five samples have also undergone DNA analysis and preliminary results indicate that the resin might be *Pistacia terebinthus* and not *Pistacia lentiscus*, due to the genetic similarity shown in their profiles to reference genomes of this species.¶¶A publication on this topic is being prepared by Lisa Briggs *et al.* Although consensus exists that distinguishing between different species of *Pistacia* is not possible based on monoterpenoids or the triterpenoid fraction,^44^ comparisons with reference samples of *Pistacia terebinthus* (not available for this study) would be interesting. The identification of monoterpenes is also extremely difficult (Table S1†), due to the high number of possible isomers,^16,52–55^ and is beyond the scope of this investigation. Nonetheless, their abundant presence is further proof of the nearly perfect preservation of the internal part of the resin lump samples KW 39, KW 49, KW 215 and KW 605. The anoxic waterlogged environment, as well as the containers and the bulk form of these samples must have played a fundamental role in such unusual preservation, which may be considered even better compared to the reference sample under investigation. This is an important point to underline, as reference samples are often commercial ones that have been, at least partially, treated,^3^ possibly resulting in the loss of some molecular components.

As an additional interesting observation, sample KW 39, which appears as the best preserved one, is yellow in colour, whereas KW 49, KW 215 and KW 605 have a more orange tone. By contrast, KW 144 is dark orange in colour, suggesting a possible correlation between the light colour of the resin and its good preservation state,^3^ although the presence of the oxidised oil cannot be ruled out as contributor of the darker colour of KW 144.^56^

The resin lumps in the Fitzwilliam Museum collection (F45A) provide an instructive comparator for this material. Although of uncertain context, their excavation origin at Abydos indicates a dry preservation environment in contrast to waterlogged condition of the Uluburun containers. Significantly, the Abydos lumps also display excellent molecular preservation with a range of triterpenoid biomarkers and abundant monoterpenes. The lumps also contain fatty acids and dicarboxylic acids consistent with a plant oil component. This reinforces the role of lump morphology in resin preservation but also provides a further example of mixed material in a bulk context. The Fitzwilliam Museum catalogue describes the lumps as resin but, in the absence of contextual information, other purposes such as raw material for decorative applications can be proposed. In either case, the advanced preparation of the oil/resin mixture is evidenced, providing an insight into the practice of material preparation.

#### Coffins – varnish and coating

**Fig. 7:**
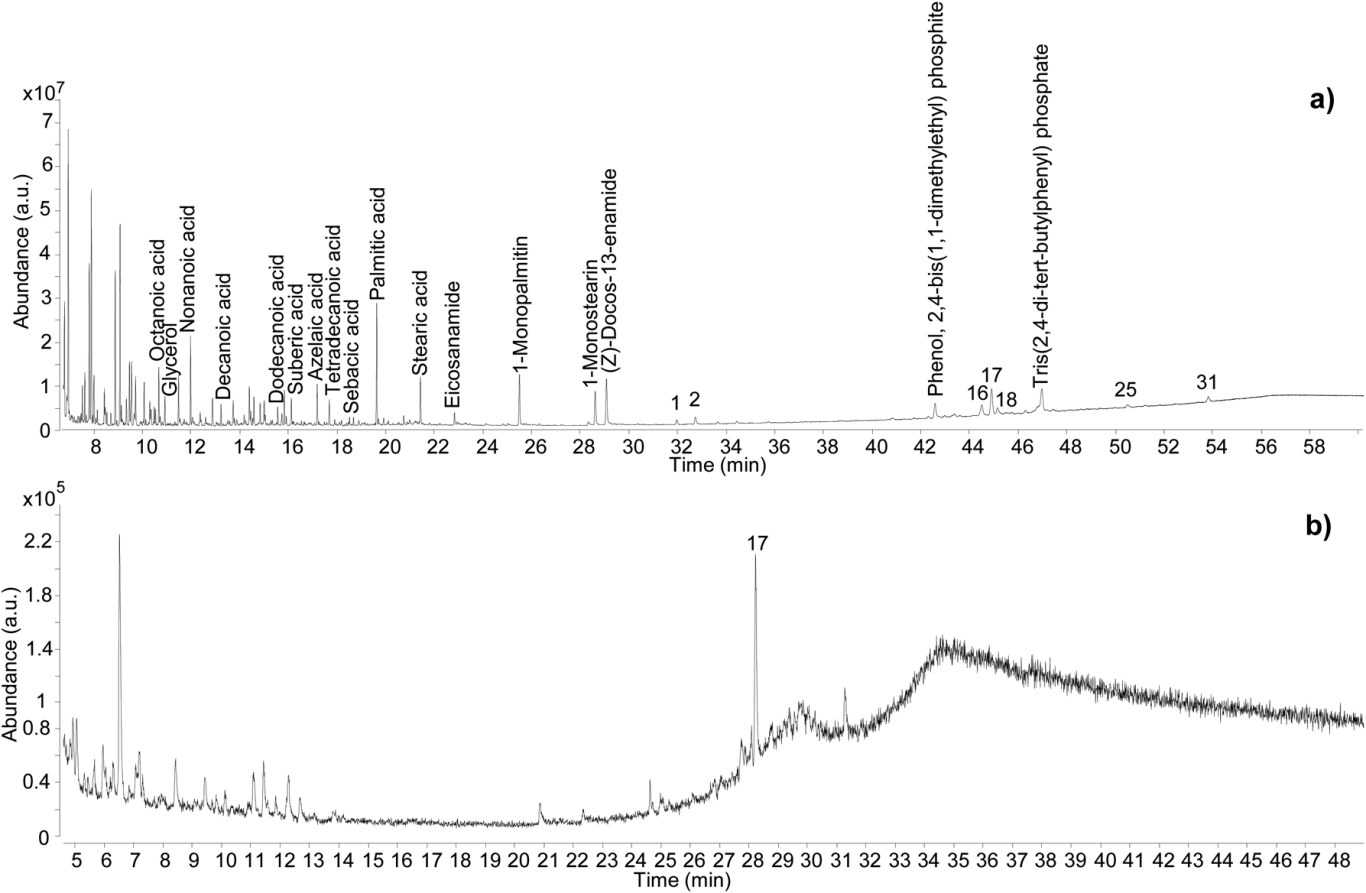
Chromatographic profiles obtained for sample C11_R1 by GC-QToF-MS (a), and by simple GC-MS (b). Numbers refer to Table 2. © The Trustees of the British Museum. Shared under a Creative Commons Attribution-NonCommercial-ShareAlike 4.0 International (CC BY-NC-SA 4.0) licence.

Varnish and coating formulations generally represent a more challenging analytical scenario compared to resin lumps. These formulations can include mixtures of organic and inorganic materials, such as pigments, which interact with each other. Moreover, such mixtures were applied as thin layers, which have large surface areas, hence greater opportunity for loss of volatiles, light-induced degradation, and exposure to oxygen. Finally, as varnishes and coatings are often associated with decorative areas, sampling constraints can be more challenging, often drastically reducing the sample sizes. These factors can make the composing materials difficult to extract and identify. However, the GC-QToF-MS analytical conditions adopted showed clear advantages compared to the GC-MS conditions used prior to the optimisation. Fig. 7 shows a comparison between the chromatographic profiles obtained for sample C11-R1 by using GC-QToF-MS and simple GC-MS.

In addition to showcasing the higher sensitivity of the GC-QToF-MS method, the results showed that the *Pistacia* resin identification in this sample was based on a few molecular markers, with 22,23,24,25,26,27-hexakisnor-dammaran-3,20-dione (1), 22,23,24,25,26,27-hexakisnor-dammaran-3-hydroxy-20-one (2), 20,24-epoxy-25-hydroxy-dammaran-3-one (16), moronic acid (17), and oleanonic acid (18) present as the most abundant ones. Although the absence of 28-norolean-17-en-3-one alone should not be taken as an indication that the resin had not undergone heating, in this case the relative abundance of moronic acid is higher compared to oleanonic acid. An opposite trend was observed during the heating experiment, with oleanonic acid proving to be more stable to heat than moronic acid. Therefore, it is reasonable to speculate that significant heating was not applied to the resin in this sample. Monopalmitin and monostearin were detected as well as some amides. These compounds were also present in the reference sample of *Pistacia* resin and in the Uluburun samples. A lipid material was also identified by detection of several even-numbered aliphatic carboxylic acids (from C8 to C18). The additional presence of dicarboxylic acids, such as sebacic, azelaic and suberic acids, suggest that a vegetable oil with drying or semi-drying properties was likely to be added to the varnish/coating formulation. Another example of the higher sensitivity and detecting capabilities of GC-QToF-MS over simple GC-MS is reported in Fig. S15 (ESI),† showing the results obtained for sample C13-R7. In this case, *Pistacia* resin markers were only detectable by GC-QToF-MS.

**Fig. 8:**
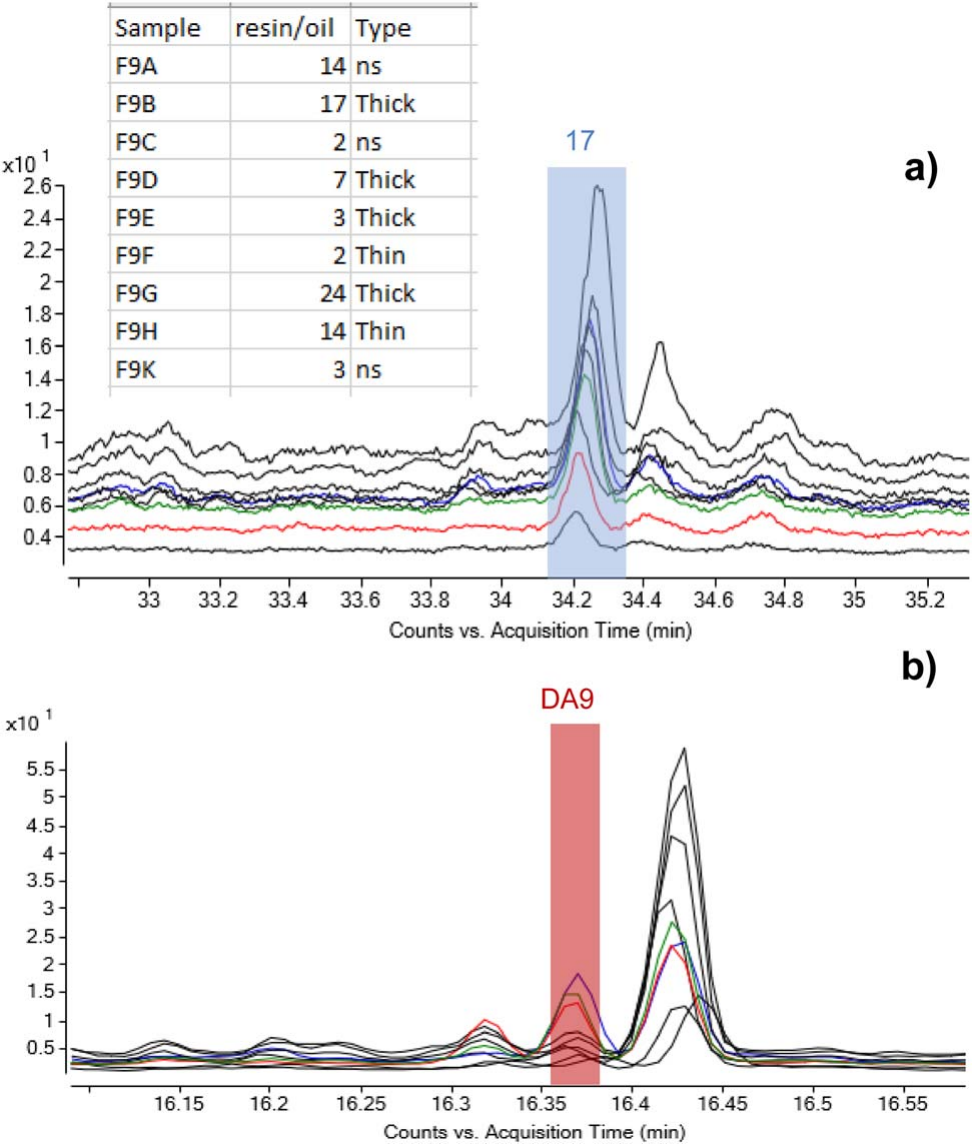
Stacked chromatograms of the nine samples from F9 highlighting the peaks corresponding to (a) moronic acid and (b) azelaic acid (DA9) and showing variability of the oil/resin ratio observed over a single object. The values in the table represent ratios of peak areas of moronic and azelaic acids with the relation to the thickness of the layers. © The Trustees of the British Museum. Shared under a Creative Commons Attribution-NonCommercial-ShareAlike 4.0 International (CC BY-NC-SA 4.0) licence.

As set out in the introduction, an ambition of this investigation was to better understand the application method of *Pistacia* resin as varnish or coating. As the material is a solid, it must be turned into a liquid or pseudo-liquid state to be applied as a thin layer, either by dissolving in some medium or by use of heat to melt it. Experiments in reproduction of coffin varnishes have demonstrated that melted resin hardens very rapidly,^57^ making it impossible to apply as a layer in a reasonable amount of time. The detection of an oil mixed with the resin suggests that a method similar to the “oil varnish” formulation previously discussed for painting practice^4^ provided the required working properties. Methods to obtain the so-called “mastic oil”, which is a simple mixture of olive oil in which some mastic tears are dissolved can also be found from cooking recipes. Other oleo–resin mixtures are relatively common, for example linseed oil and colophony.^58^ Such mixtures have been successfully used to reproduce Egyptian varnishes experimentally.^57^ Therefore, it is significant that the detection of mastic resin markers accompanied by drying/semi-drying oil markers was a common result for a large number of varnishes taken from 22nd Dynasty coffins from the British Museum's collection. In addition to C11-R1 and C13-R7 already mentioned, these samples include C3-R2, C2_R1, C4_R2, C3_R1, C11_R2, B8_R1 and B8_R2 (Table 1). The extensive application of this varnish formulation in ancient Egyptian coffin manufacture is further supported by results previously acquired from varnishes, coatings and residues on Egyptian coffins in the collection of the Fitzwilliam Museum. The original study examined some 100 samples from more than 50 objects of dates ranging from the New Kingdom (c.1550 BC–c.1069 BC) until the Roman period (30 BC – 641 AD).^46,59^ Around 30% of the samples contained terpenoid markers of *Pistacia* resin,||||Detailed discussion of the wider Fitzwilliam Museum data corpus is beyond the scope of this article and will be the subject of a separate publication. and the most relevant examples are listed in Table 1. Significantly, around 80% of these *Pistacia*-containing samples also contained mono- and dicarboxylic fatty acids, indicating the presence of partially oxidised oil. A summary of the results obtained for these samples is reported in Table S2 (ESI).† Notably, only one *Pistacia*-containing sample described as a ‘varnish’ contained no oil (F30A), and most of the yellow/golden coatings exhibited a mixture of *Pistacia* resin and oil. Among the few other samples without oil, one contained another type of unidentified fat (F52B), and one contained beeswax (F46A), indicating alternative mixtures with the resin, although the context of some of these substances is more ambiguous and many are black. Beeswax (F43A) and conifer resin (F28A) were also seen included in mixtures with *Pistacia* and oil. Notwithstanding these variations, the results overall show a consistent mixing technology for yellow-coloured varnishes and support the hypothesis that a lipid material, in most cases an oil, was used as medium to dissolve and apply the resin as a liquid. This mixture would enable targeted and precise application to specific areas of coffin decoration, as is often observed on the surface of coffins. It would also have drying properties that would allow for it to cure into a layer of variable thickness depending on the resin/oil ratio. The nine samples taken from different areas of the Nesperwershefyt coffin (F9A-K) set provide an instructive example illustrating the variability of the ratio of resin-derived moronic acid and oil-derived azelaic acid (by peak area) in varnish layers of different thicknesses (Fig. 8). The thicker layers tend to indicate richer resin composition, as might be expected. The correlation, however, is not consistent and differential preservation factors in thin and thick varnish films will add complexity to interpretation of such a simple ratio.

It must also be highlighted that the extraction method used in all these analyses is a simple solubilisation in DCM. Such a method is suitable for the solubilisation of free and poorly bound molecules, including resin components and lipid components that had undergone hydrolysis during ageing and are not bound in a polymeric network. DCM does not have any saponification power, which would be needed to have a more realistic idea of the relative amounts of the materials present in the mixture. Moreover, these analyses were conducted with method A and could not be repeated using GC-QToF-MS due to lack of residual samples. As a result, in those few samples in which markers of oil were not detected, the absence of oil cannot be assumed.

### Residues on ceramics (likely incense burners)

**Fig. 9:**
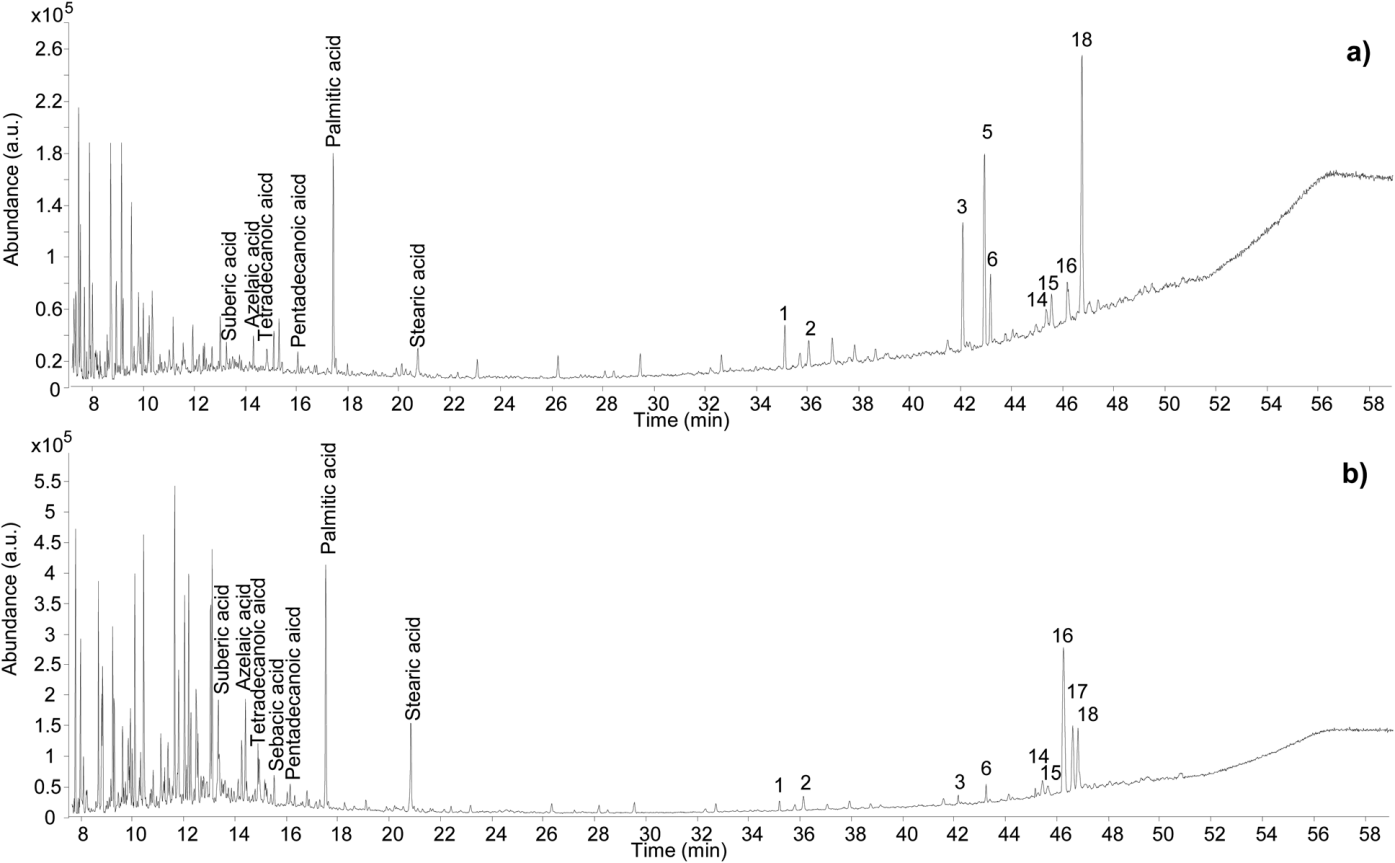
Chromatographic profiles obtained for samples Sai 012 (a) and Sai 0245 (b). Numbers refer to Table 2. © The Trustees of the British Museum. Shared under a Creative Commons Attribution-NonCommercial-ShareAlike 4.0 International (CC BY-NC-SA 4.0) licence.

Different chromatographic profiles were obtained for samples Sai 012 and Sai 0245 (Fig. 9), which were taken from residues on ceramic sherds likely to have been used as incense burners. Although the results for these samples again reveal the presence of *Pistacia* resin and oil, the distribution of the compounds, especially the resin markers, was different. For Sai 012, oleanonic acid (18) was detected, but not moronic acid (17). Additionally, 28-norolean-17-en-3-one (5) was present with high relative abundance. These observations agree with the results obtained in our heating experiment, suggesting a substantial heating treatment of the oleo-resin mixture. For Sai 0245, moronic and oleanonic acids were detected with comparable abundances and no 28-norolean-17-en-3-one was present. 20,24-Epoxy-25-hydroxy-dammaran-3-one (16) was present with particularly high abundance. Evidence for a harsh heat treatment was therefore not obtained for this sample. Additionally, sample Sai 0245 appears much lighter in colour compared to sample Sai 012 (Fig. S2, ESI†), which could be linked to the exposure to heat. Nevertheless, it appears from the results obtained in this investigation that interpretations around colour change are difficult to draw. Heating, mixing of multiple materials potentially subject to darkening, and the possible presence of pigments are only some of the factors that create a very complex scenario around the significance of colour in this context.

## Conclusions

This study presents the first application of an optimised method based on GC-QToF-MS analysis for the enhanced characterisation of mastic (*Pistacia* sp.) resin and its identification in archaeological samples. The method showcased various advantages compared to simple GC-MS methods routinely used for the analysis of resins in the cultural heritage field. Enhanced sensitivity and structural elucidation capabilities enabled additional molecular components to be observed and resin markers to be identified even in complex archaeological samples.

Additionally, the formation of 28-norolean-17-en-3-one was proved to occur upon heating not only in anoxic conditions,^6^ but also in an open air environment, strengthening its possible use as a heating marker of the resin in historical objects. Moronic acid appeared to degrade upon heating, whereas oleanonic acid appeared to be more stable, making the relative abundance of these two acids an additional indication of thermal degradation. However, 28-norolean-17-en-3-one further degrades above 250 °C and upon longer exposure to heat, similarly to most triterpenoid molecules. Therefore, its absence cannot in principle be considered evidence that the resin was not heated.

The analytical results suggest that, for the extremely well-preserved resin samples from the Uluburun shipwreck, the jars might have been filled with solid resin that might have partially melted naturally under the sun heat. By contrast, the results from a large number of archaeological samples from Egyptian coffins in varnish/coating formulations showed that *Pistacia* resin was often mixed with a lipid material, in most cases a drying/semi-drying oil, which was used as a medium to dissolve the solid resin over a gentle heat to apply it as a layer of variable thickness. Additional studies by GC-QToF-MS are foreseen to enhance the identification of monoterpenoids, when present, to help clarify the possible use of essential oils in similar formulations. Furthermore, the study of chemical and physical interactions between *Pistacia* resin and drying/semi-drying oils emerges as a research area of particular interest. The creation of mock-up samples and attention to possible co-polymerisation markers^58^ might provide additional proof of the technology behind the use of *Pistacia* resin in ancient times.

## Author contributions

Conceptualisation: DT, RS; data curation: DT, RS; formal analysis: DT, KF, LB, NvA, RS; funding acquisition: RS; investigation: DT, RS; DT, KF, LB, NvA, RS; methodology: DT, KF, NvA, RS; resources: DT, KF, LB, CP, RS; supervision: DT, RS; validation: DT; visualisation: DT, RS; writing – original draft: DT; writing – review and editing: DT, KF, LB, NvA, CP, RS.

## Conflicts of interest

There are no conflicts to declare.

## Supplementary Material

RA-014-D3RA06651G-s001
