# Supplementary material for: Advances in the characterisation and identification of mastic (Pistacia sp.) resin in archaeological samples by GC-QToF-MS
Source: RSC Adv. 2024 Jan 2;14(2):836–54. doi: 10.1039/d3ra06651g (PMC10759165; doi:10.1039/d3ra06651g)
Supplement: RA-014-D3RA06651G-s001 [file RA-014-D3RA06651G-s001.pdf]

Electronic Supplementary Material (ESI) for RSC Advances.
This journal is © The Royal Society of Chemistry 2023

Supplementary Information

Advances in the study of mastic (*Pistacia* sp.) resin composition, use and heat-induced alteration in archaeological contexts by gas chromatography coupled to high resolution and high accuracy mass spectrometry

Diego Tamburini[1]*, Kate Fulcher[1], Lisa Briggs[1,2], Nelly von Aderkas[1], Cemal Pulak[3], Rebecca Stacey[1]

[1] Department of Scientific Research, The British Museum, Great Russell Street, London WC1B 3DG, UK

[2] Cranfield University, College Rd, Cranfield, Wharley End, Bedford MK43 0AL, United Kingdom

[3] Institute of Nautical Archaeology at Texas A&M University; Department of Anthropology, Texas A&M University, College Station, Texas, United States

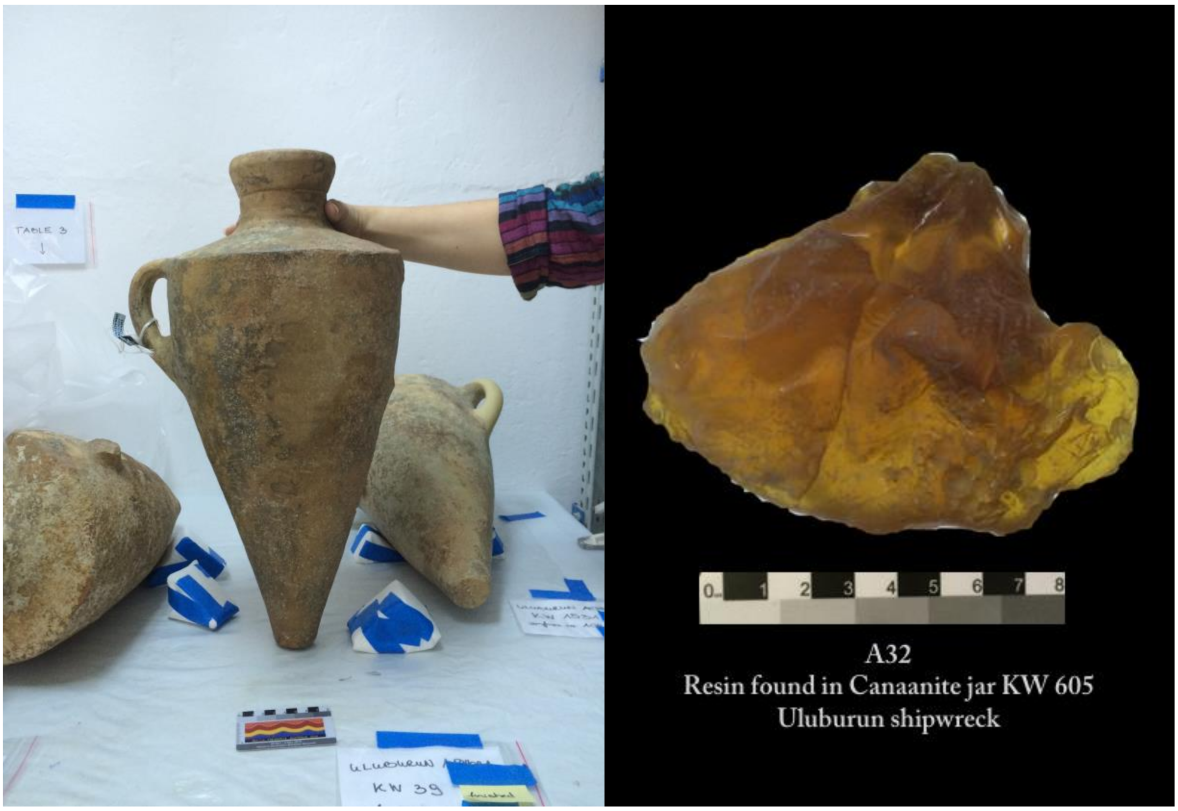

Figure S1. Images of Canaanite jar KW 39 (left) and resin sample KW 605 (right). The sample designations are derived from the number ascribed to each Canaanite jar; for example, Canaanite jar KW 39 yielded resin sample KW 39, and so on.

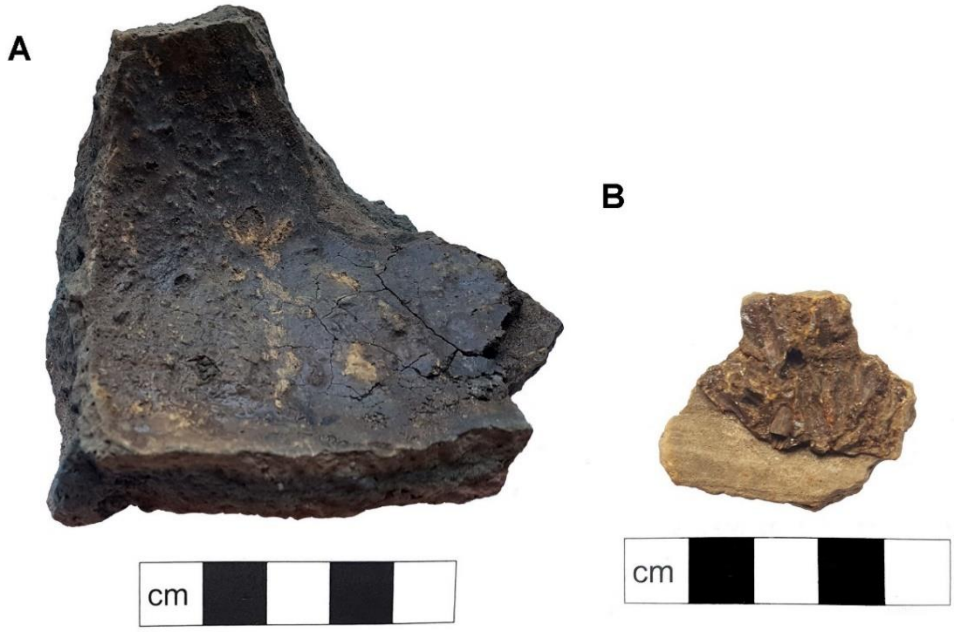

Figure S2. Images of the incense burners from Sai. Sample Sai 012 was taken from A and sample Sai 0245 was taken from B. © The Trustees of the British Museum. Shared under a Creative Commons Attribution-NonCommercial-ShareAlike 4.0 International (CC BY-NC-SA 4.0) licence.

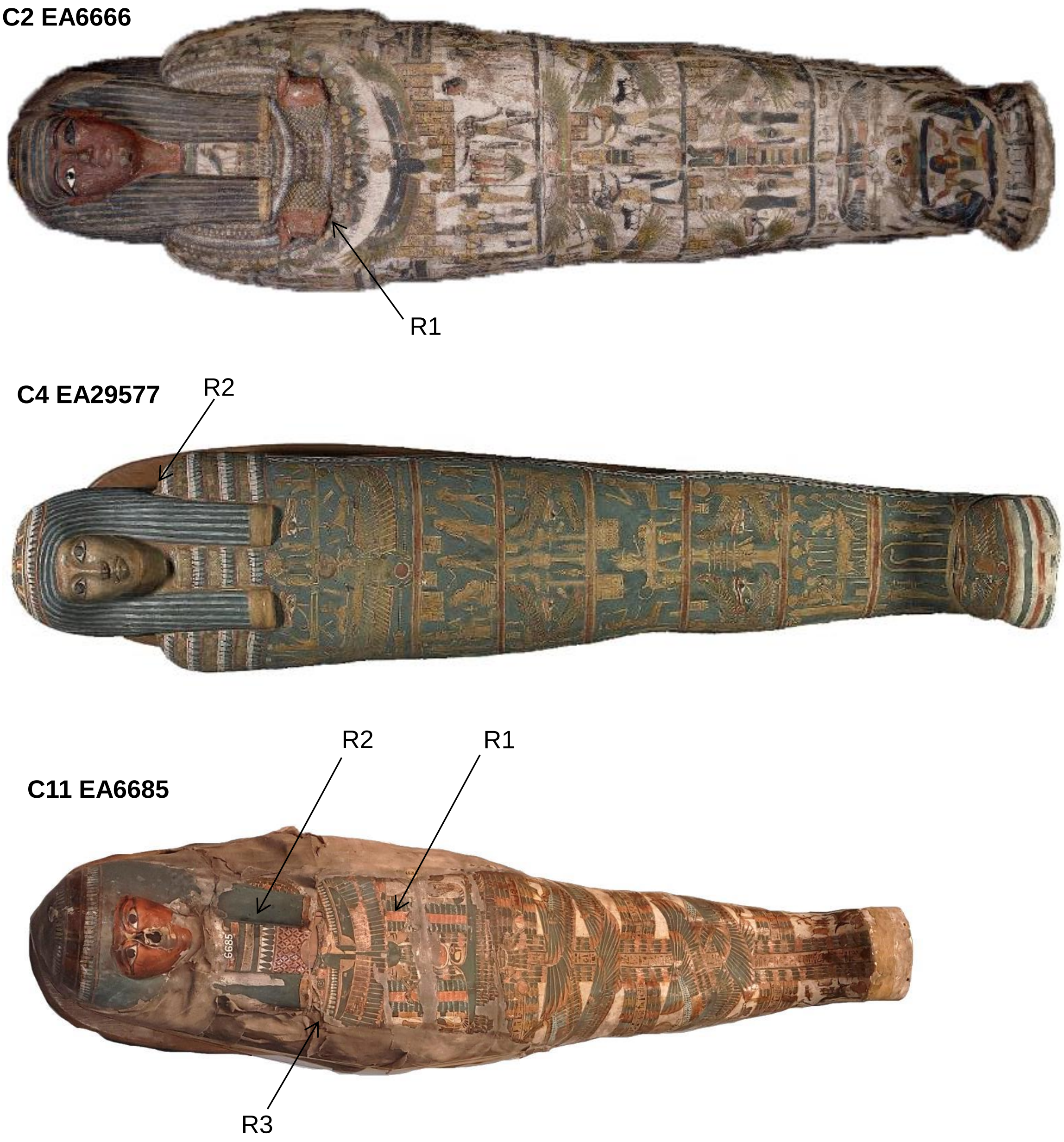

Figure S3. Images of the coffins from the British Museum's collection and sampling locations.
© The Trustees of the British Museum. Shared under a Creative Commons Attribution-NonCommercial-ShareAlike 4.0 International (CC BY-NC-SA 4.0) licence.

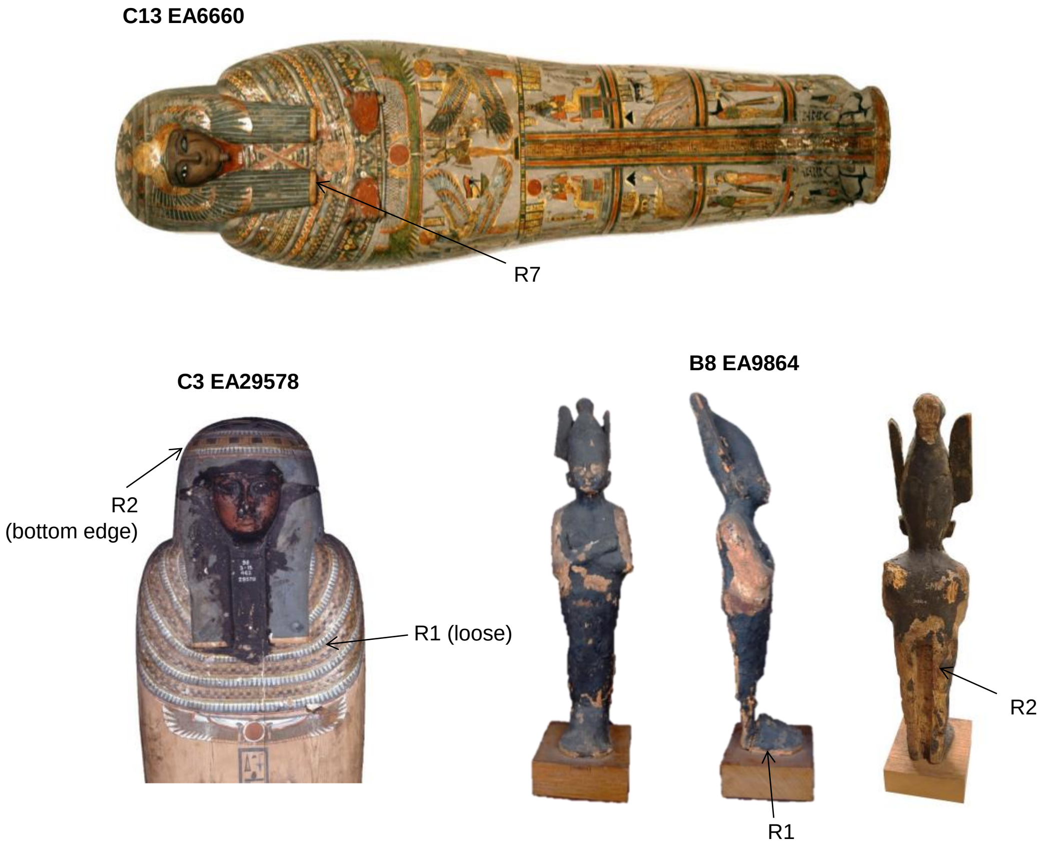

Figure S4. Images of the coffins/objects from the British Museum's collection and sampling locations. © The Trustees of the British Museum. Shared under a Creative Commons Attribution-NonCommercial-ShareAlike 4.0 International (CC BY-NC-SA 4.0) licence.

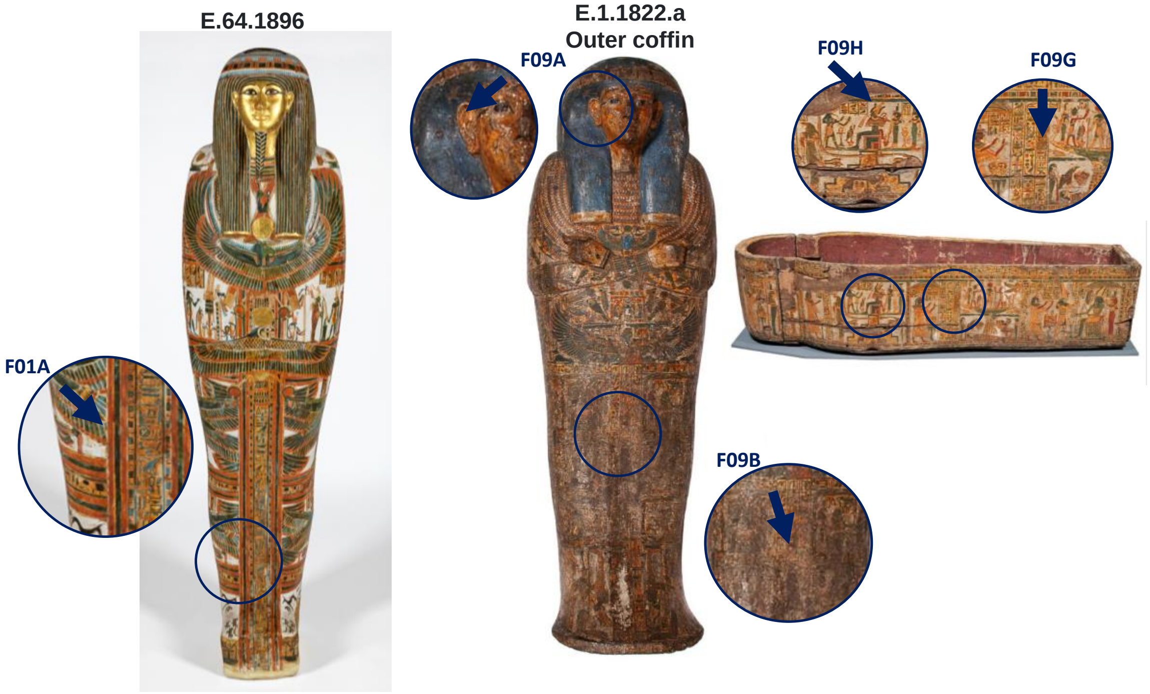

Figure S5. Images of the coffins from the Fitzwilliam Museum's collection and sampling locations. © Fitzwilliam Museum.

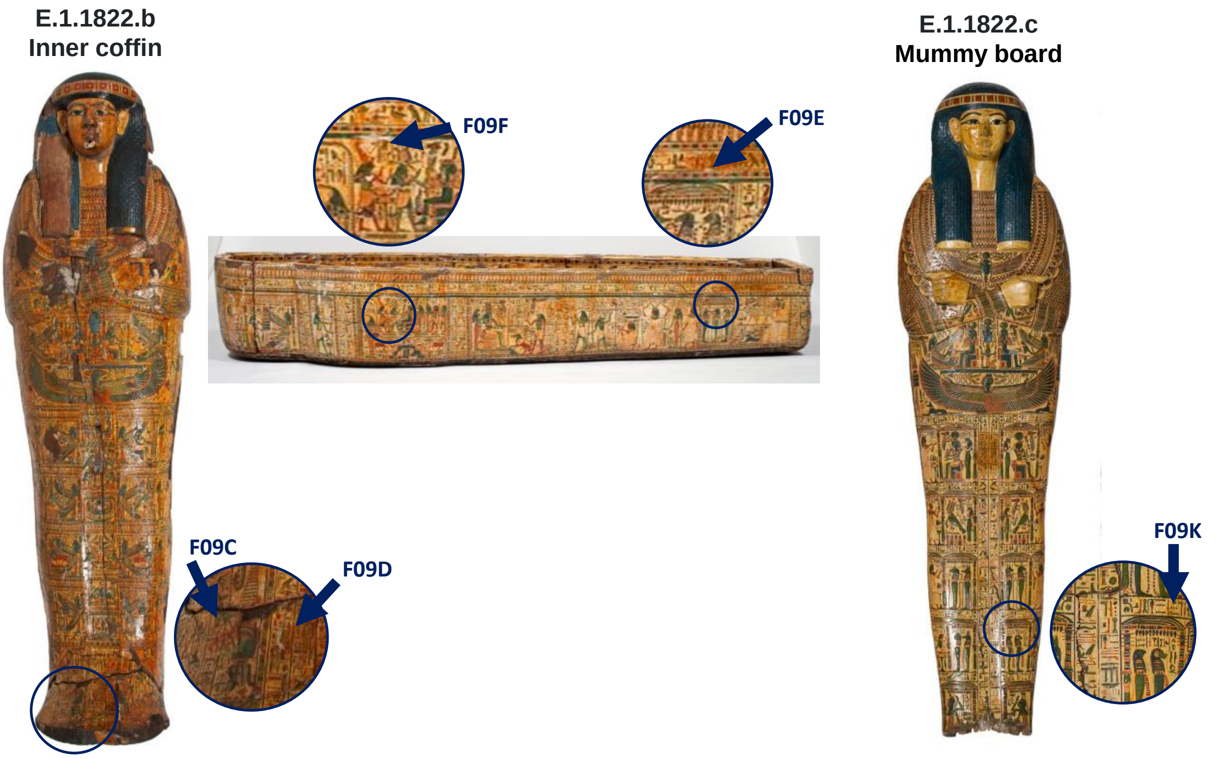

Figure S6. Images of the coffins from the Fitzwilliam Museum's collection and sampling locations. © Fitzwilliam Museum.

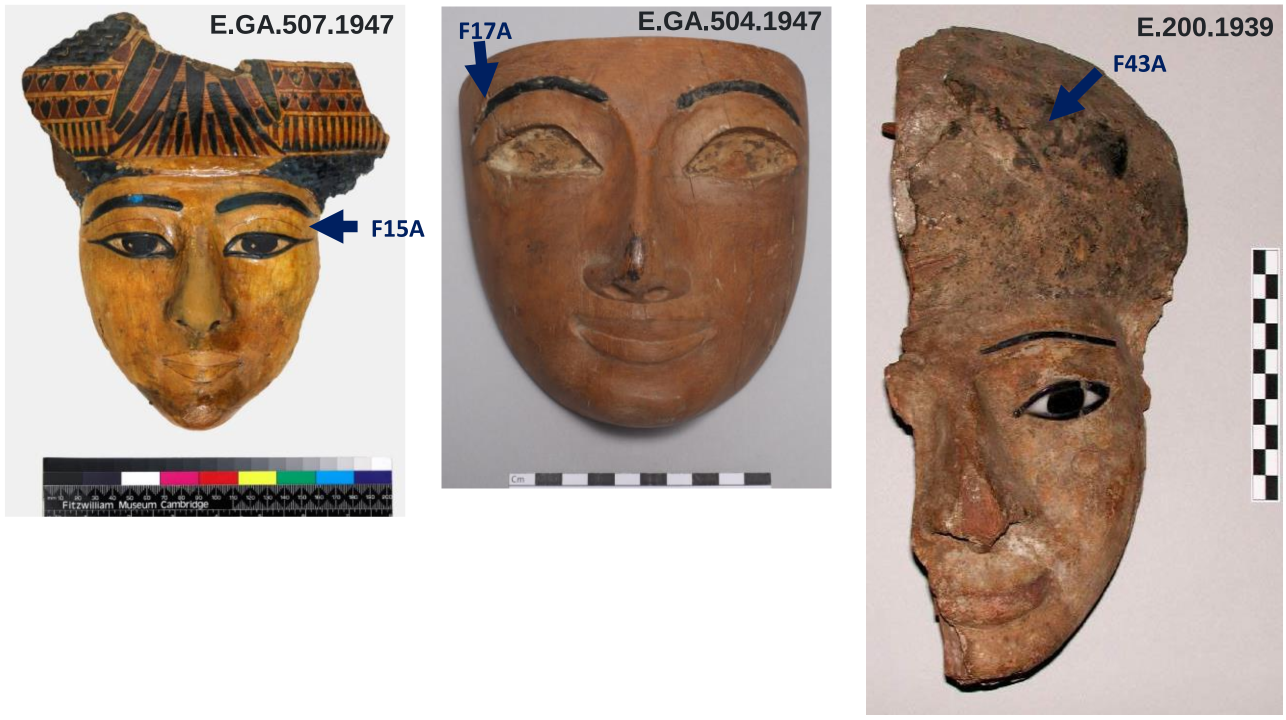

Figure S7. Images of the coffins (faces) from the Fitzwilliam Museum's collection and sampling locations. © Fitzwilliam Museum.

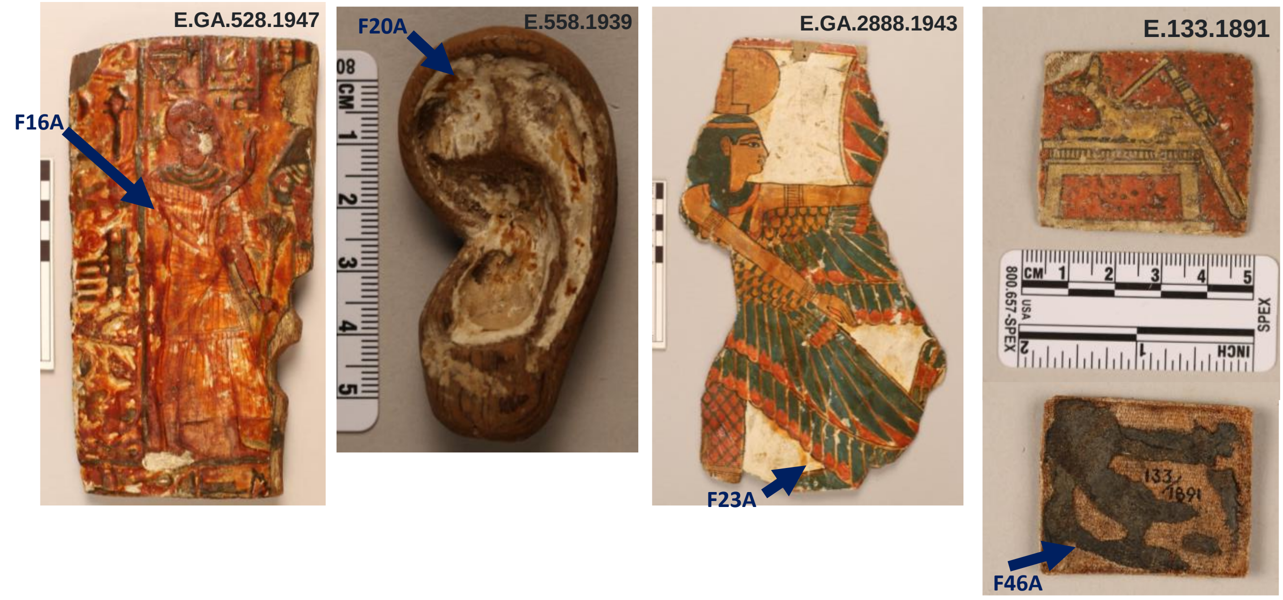

Figure S8. Images of coffin and cartonnage fragments from the Fitzwilliam Museum's collection and sampling locations. © Fitzwilliam Museum

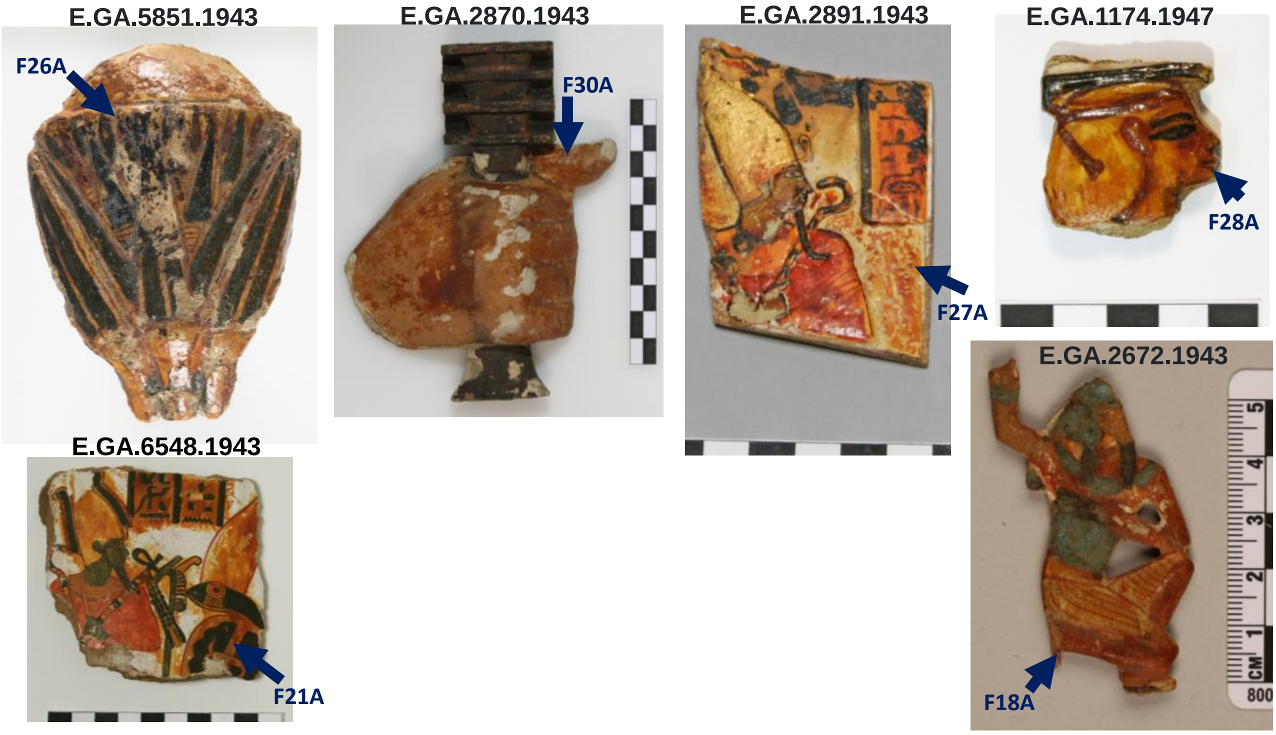

Figure S9. Images of coffin/cartonnage fragments from the Fitzwilliam Museum's collection and sampling locations. © Fitzwilliam Museum

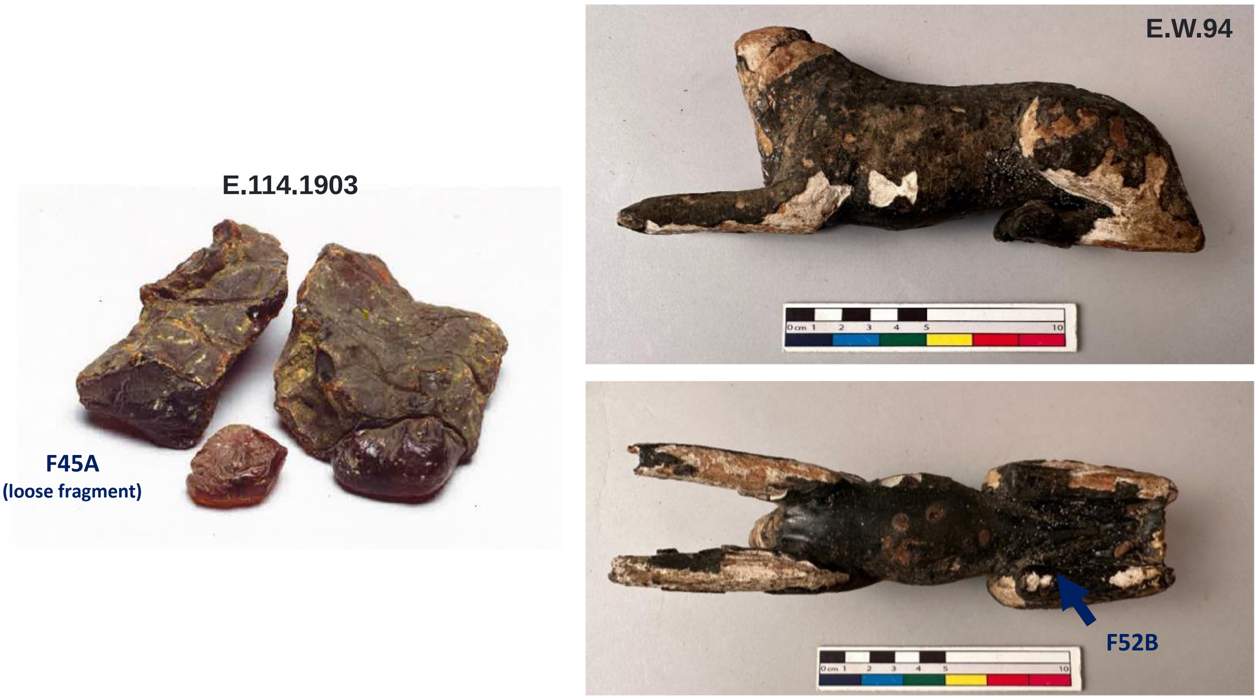

Figure S10. Images of resin lump (E.114.1903) and jackal figure (E.W.94) from the Fitzwilliam Museum's collection and sampling locations. © Fitzwilliam Museum

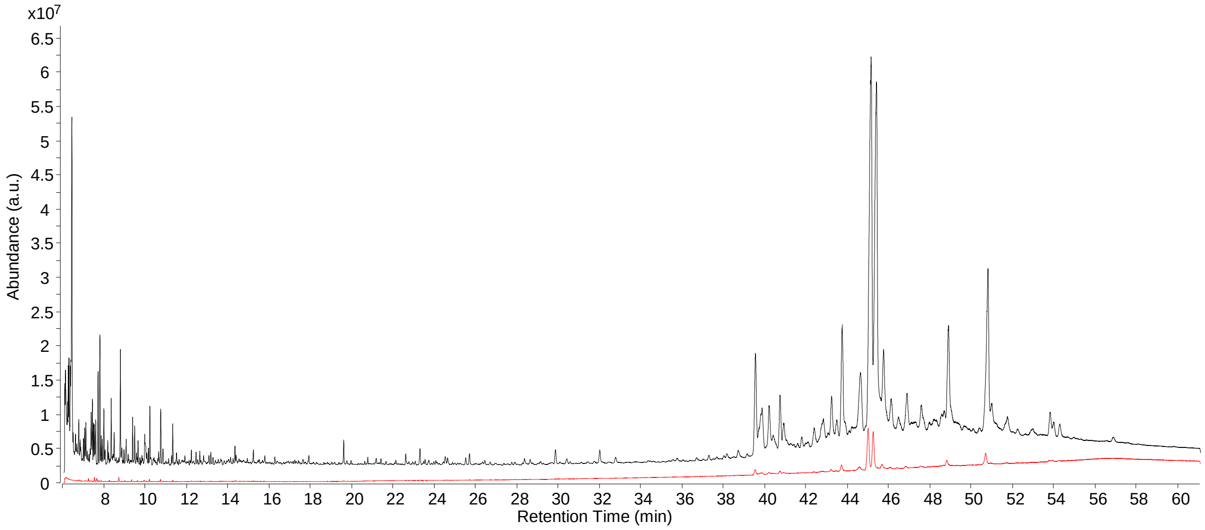

Figure S11. Comparison between the chromatograms obtained for *Pistacia* resin in splitless mode (black) and 1:10 split ratio (red). © The Trustees of the British Museum. Shared under a Creative Commons Attribution-NonCommercial-ShareAlike 4.0 International (CC BY-NC-SA 4.0) licence.

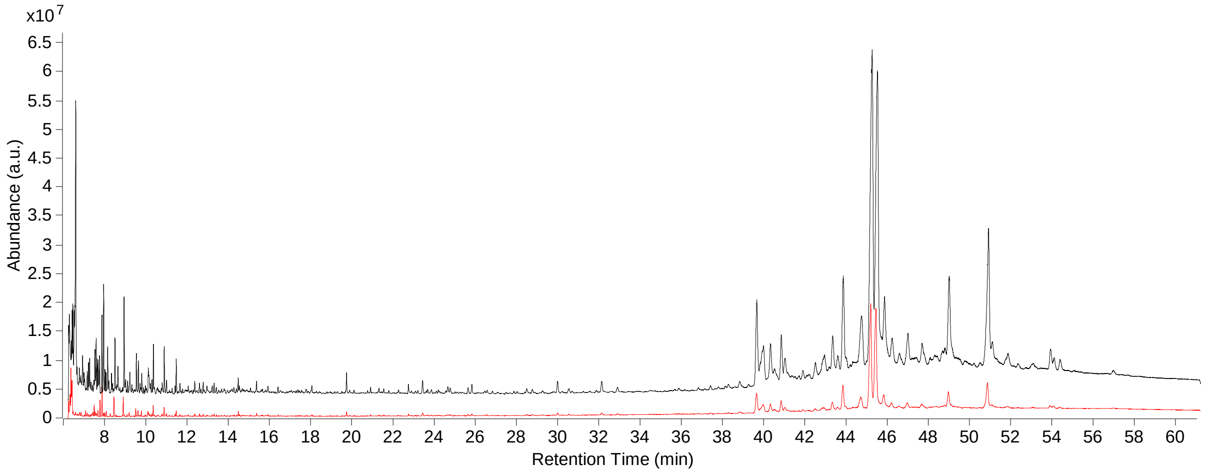

Figure S12. Comparison between the chromatograms obtained for *Pistacia* resin in standard EI mode (black) and low EI mode (red). © The Trustees of the British Museum. Shared under a Creative Commons Attribution-NonCommercial-ShareAlike 4.0 International (CC BY-NC-SA 4.0) licence.

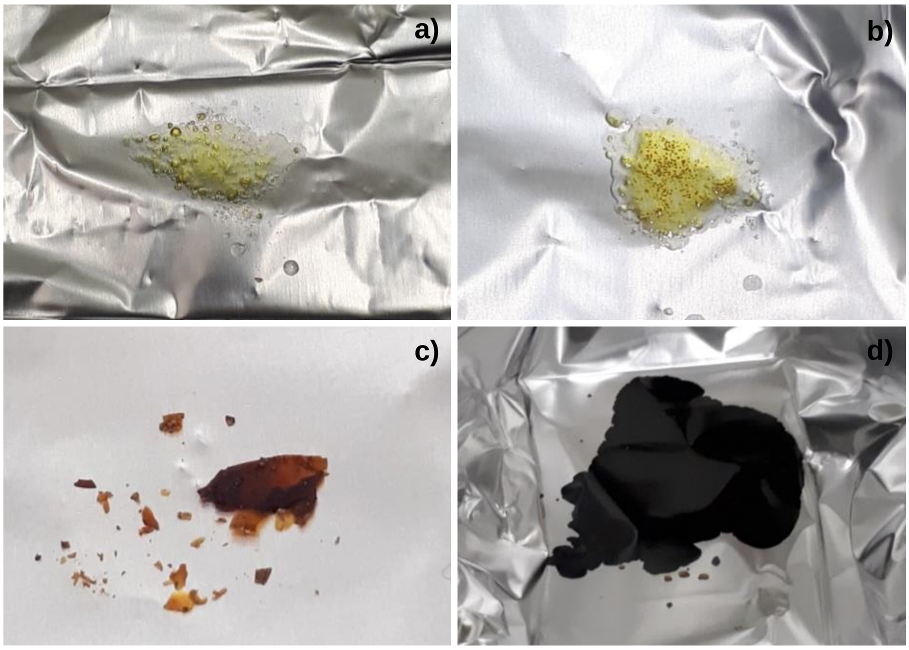

Figure S13. Images showing the differences in appearance of the *Pistacia* reference samples after the heating experiment: **a)** 100°C for 4 hours; **b)** 100°C for 8 hours; **c)** 250°C for 2.5 hours; **d)** 400°C for 10 mins. © The Trustees of the British Museum. Shared under a Creative Commons Attribution-NonCommercial-ShareAlike 4.0 International (CC BY-NC-SA 4.0) licence.

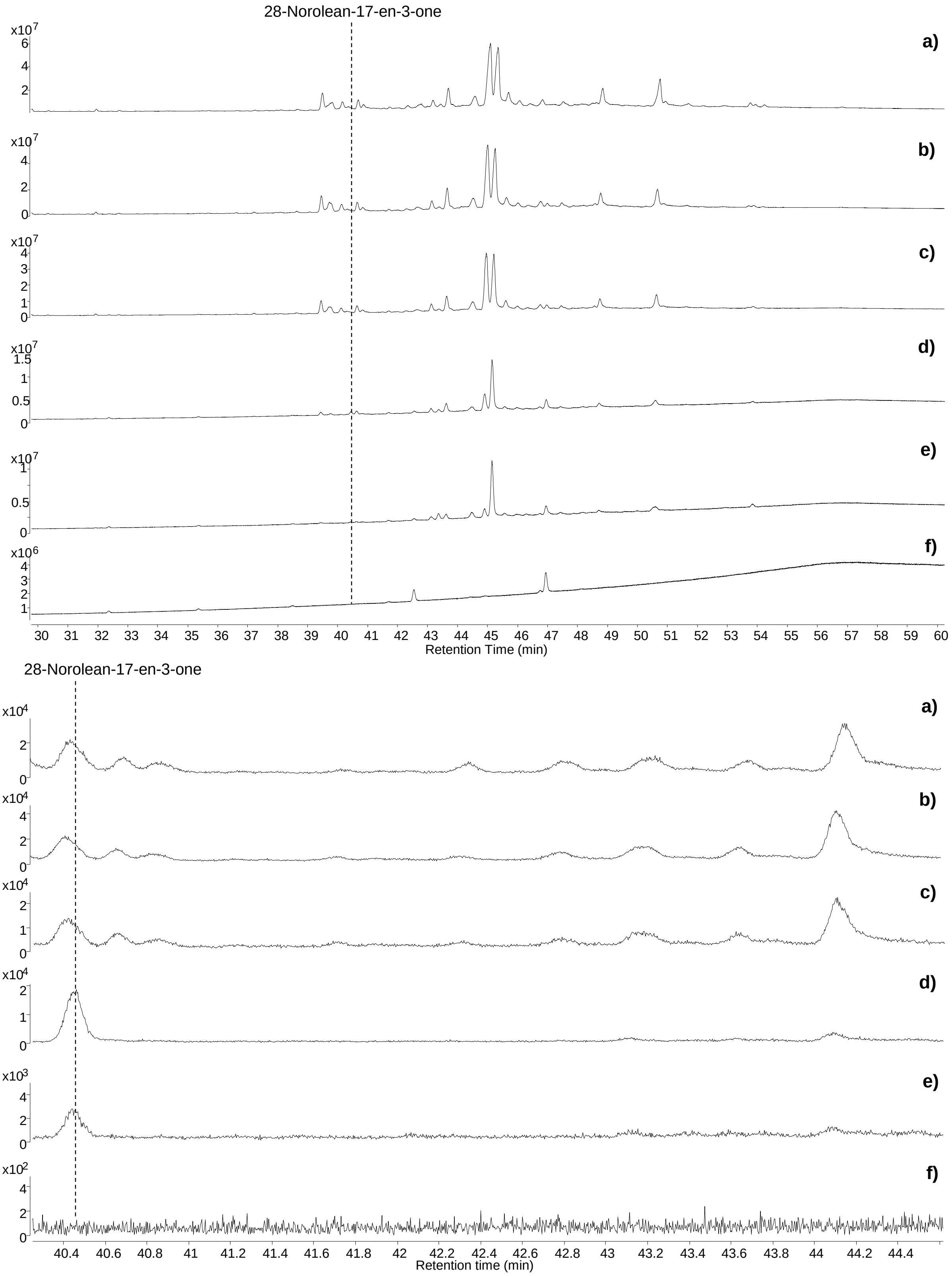

Figure S14. Expanded view (top) and extracted ion chromatograms (bottom) of *m/z* 410.3350 for the profiles obtained by GC-QToF analysis of the *Pistacia* reference sample (**a**) and the samples from the heating experiment at 100°C for 4 hours (**b**)**,** 100°C for 8 hours (**c**)**,** 250°C for 0.5 hours (**d**), 250°C for 2.5 hours (**e**), 400°C for 10 mins (**f**). © The Trustees of the British Museum. Shared under a Creative Commons Attribution-NonCommercial-ShareAlike 4.0 International (CC BY-NC-SA 4.0) licence.

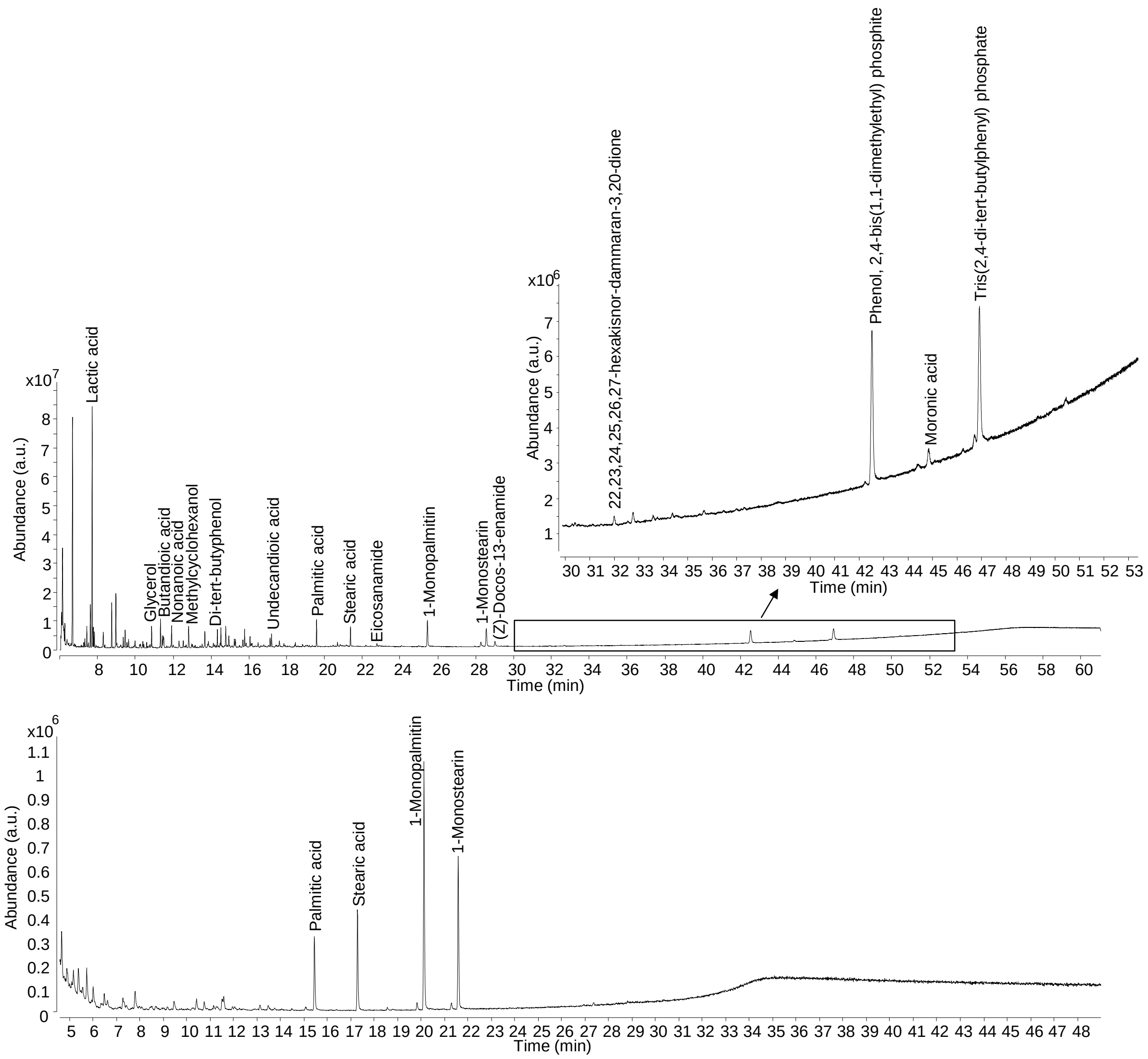

Figure S15. Comparison between the chromatograms obtained for sample C13-R7 by using GC-QToF and optimised conditions (**top**), and standard GC-MS with non-optimised conditions (**bottom**). The main compounds are indicated, and an expansion of the top chromatogram is provided, showing *Pistacia* resin markers not detected in the bottom chromatogram. © The Trustees of the British Museum. Shared under a Creative Commons Attribution-NonCommercial-ShareAlike 4.0 International (CC BY-NC-SA 4.0) licence.

Table S1. Compounds detected in the Uluburun samples not listed in Table 2. The monoterpenoids exact structures are difficult to assign in most cases without standards. This was beyond the scope of this article, and we refer to the available literature [1-5].

Nº	Compound	Retention time	Raw formula	Calculated *m/z**	Fragment ions*
a	1-methyl-4-(1-methylethenyl)-benzene	8.27	$C_{10}H_{12}$	132.0934	**117.0697**, 115.0541, 91.0541, 65.0385
b	monoterpene 1	8.55	$C_{10}H_{16}O$	152.1196	137.0961, 119.0855, 109.1010, 95.0491, 91.0542, 82.0776, 79.0541, **67.0541**, 55.0542
c	bornanone	9.11	$C_{10}H_{16}O$	152.1196	143.0521, 108.0931, **95.0854**, 81.0696, 67.0540, 55.0541
d	monoterpene 2	9.62	$C_{10}H_{14}O$	150.1039	**135.0879**, 119.0488, 105.0697, 91.0540, 65.0384
e	4,6,6-trimethyl-bicyclo-hept-3-en-2-one	10.00	$C_{10}H_{14}O$	150.1039	135.0803, 122.1087, **107.0855**, 91.0541, 79.0540, 67.541
f	monoterpene 3 -TMS	10.24	$C_{13}H_{24}OSi$	224.1591	209.1354, 181.1042, 165.0728, **144.0990**, 129.0728, 119.0852, 105.0697, 91.0540, 73.0467
g	monoterpene 4 -TMS	10.77	$C_{13}H_{24}OSi$	224.1591	209.1356, 181.1043, **155.0894**, 134.1088, 119.0853, 101.0415, 92.0617, 91.0541, 73.0472
h	monoterpene 5 -TMS	11.31	$C_{13}H_{22}OSi$	222.1434	**207.1274**, 191.0886, 177.0728, 133.1008, 117.0697, 105.0697, 91.0540, 73.0467
i	monoterpene 6 -TMS	12.19	$C_{13}H_{26}O_2Si$	242.1697	227.1095, 193.0677, 165.0728, 149.0957, **133.1063**, 119.0489, 105.0696, 91.0540, 73.0672, 65.0384
j	monoterpene 7 -TMS	12.24	$C_{13}H_{24}O_2Si$	240.1540	225.1303, 150.1038, 117.0364, **108.0932**, 93.0697, 73.0466
k	cuminyl alcohol (?)	12.43	$C_{13}H_{22}OSi$	222.1432	207.1197, 179.0885, 165.0729, **133.1010**, 117.0696, 105.0697, 96.0479, 91.0540, 73.0466
l	myrtenoic acid	12.64	$C_{13}H_{22}O_2Si$	238.1384	223.1148, 195.0832, 179.0521, 148.0880, 133.0646, 105.0696, 91.0541, **73.0467**
m	monoterpene 8 -TMS	13.08	$C_{13}H_{22}O_2Si$	238.1384	223.1149, 195.0835, 167.0886, 151.0573, 144.0963, 133.0646, 119.0854, 105.0697, 91.0541, **73.0466**
n	monoterpene 9 -TMS	13.47	$C_{13}H_{22}O_2Si$	238.1384	223.1148, 205.1043, 181.1043, **135.0802**, 91.0542, 73.0467
o	monoterpene 10 -TMS	13.98	$C_{13}H_{20}O_2Si$	236.1227	**221.1020**, 202.1715, 193.0677, 177.1093, 147.0801, 132.0929, 119.0853, 103.0374, 91.0540, 73.0466

p	monoterpene 11 - TMS	14.39	$C_{13}H_{20}O_3Si$	252.1176	237.0940, 224.1226, 209.0990, 193.0679, 183.0835, 167.0522, 162.0674, 134.0724, 119.0490, 106.0775, 91.0541, **73.0467**
q	monoterpene 12 - TMS	15.22	$C_{16}H_{30}O_3Si_2$	326.1728	311.1495, 283.1181, 255.1230, 236.1223, 221.0627, 207.1199, 193.1040, 177.0729, 144.0963, 119.0852, 91.0541, **73.0466**
r	monoterpene 13 - TMS	15.86	$C_{13}H_{20}O_3Si$	252.1176	**237.1003**, 221.0628, 193.1040, 179.0520, 163.0751, 147.0438, 120.0566, 111.0349, 105.0332, 91.0541, 73.0466
s	Unknown 370_1 (2TMS)	16.99	$C_{20}H_{26}O_3Si_2$	370.1415	355.1212, 342.1677, 327.1441, **309.1337**, 252.1176, 219.0835, 192.0964, 177.0730, 147.0439, 115.0543, 91.0541, 73.0466
t	Unknown 370_2 (2TMS)	17.45	$C_{20}H_{26}O_3Si_2$	370.1415	355.1213, 325.1288, **235.1147**, 221.0629, 207.0835, 193.0315, 177.0730, 147.0440, 91.0541, 73.0467
u	Tetracyclic terpenoid	24.8	$C_{24}H_{38}$	326.2968	314.2606, 299.2371, 271.2419, 229.1951, 219.1742, 205.1588, 189.1637, 175.1480, 161.1324, 147.1167, 133.1011, 121.1012, 108.0932, **95.0855**, 79.0542, 67.0542, 55.0542
v	Tetracyclic terpenoid	25.61	$C_{22}H_{36}O$	316.2761	301.2528, 283.2421, **205.1587**, 189.1638, 163.1117, 149.1323, 135.1166, 121.1010, 108.0931, 95.0854, 81.0698, 67.0542, 55.0542
w	Tetracyclic terpenoid	26.24	$C_{25}H_{46}OSi$	390.3312	375.3077, 300.2811, 285.2578, **261.2570**, 217.1950, 205.1949, 189.1637, 177.1637, 163.1480, 149.1323, 135.1166, 123.1166, 109.1009, 95.0853, 81.0697, 73.0466, 67.0541, 55.0541
x	Tetracyclic terpenoid	30.29	$C_{28}H_{48}O_2Si$	444.3418	429.3180, 356.2711, 341.2475, 330.2554, 323.2370, 275.2367, 257.2261, 205.1586, 189.1636, 169.1036, 161.1323, 147.1166, 135.1166, 121.1010, 107.0853, **97.0647**, 81.0697, 73.0467, 67.0541, 55.0541
y	Tetracyclic terpenoid	30.55	$C_{28}H_{54}O_2Si_2$	478.3657	463.3428, 388.3155, 349.2920, 283.2421, 259.2420, 189.1638, 183.1200, **169.1044**, 147.1167, 135.1167, 129.0728, 121.1011, 107.0855, 95.0855, 81.0698, 73.0468
z	Tetracyclic terpenoid	31.05	$C_{29}H_{52}O_2Si$	460.3731	445.3490, 430.3254, 415.3027, 389.2874, 347.2768, 325.2526, 314.2602, 299.2368, **274.2292**, 257.2265, 231.1744, 219.1741, 201.1636, 187.1482, 161.1323, 147.1167, 129.0727, 121.1011, 107.0855, 95.0854, 81.0698, 73.0467, 67.0541, 55.0542
α	Tetracyclic terpenoid	33.57	$C_{26}H_{44}O_3Si$	432.3054	417.2827, 342.2559, 333.2243, 314.2596, 299.2371, 243.1744, 229.1947, 215.1794, 205.1587, 187.1481, 161.1324, 147.1168, **129.0366**, 121.1011, 109.1010, 95.0854, 81.0698, 75.0260, 67.0542, 55.0542

β	Tetracyclic terpenoid	34.36	$C_{29}H_{54}O_3Si_2$	506.3606	491.3379, 449.3264, 416.3110, 401.2871, **377.2870**, 333.2244, 319.2087, 287.2372, 259.2423, 224.1227, 203.1794, 189.1639, 177.1636, 161.1324, 135.1166, 129.0366, 121.1011, 109.1011, 95.0855, 73.0467, 55.0542
γ	Triterpenoid – isomastica skeleton	38.58	$C_{33}H_{58}OSi$	498.4263	483.4024, **393.3518**, 311.2734, 269.2263, 255.2107, 241.1951, 227.1795, 215.1793, 201.1637, 189.1637, 173.1325, 159.1167, 145.1013, 135.1167, 121.1011, 109.1011, 95.0854, 81.0697, 73.0467, 69.0698, 55.0542
δ	Triterpenoid	41.43	$C_{36}H_{64}O_2Si_2$	584.4439	569.4207, 498.4260, 482.3945, 467.3708, 388.3159, 398.2653, 279.2135, 229.1951, 203.1793, 190.1713, 175.1481, 161.1324, 147.1168, **136.1246**, 129.0725, 121.1011, 107.0855, 91.0698, 81.0697, 73.0467, 69.0698, 55.0542
ε	Triterpenoid – isomastica skeleton	41.83	$C_{36}H_{66}O_2Si_2$	586.4601	571.4371, 498.4257, 483.4024, **393.3521**, 255.2106, 241.1951, 229.1950, 215.1793, 201.1637, 187.1481, 173.1323, 159.1167, 147.1168, 135.1166, 119.0854, 109.1011, 95.0855, 81.0698, 75.0260, 69.0698, 55.0542
ζ	Triterpenoid	44.06	$C_{33}H_{56}O_2Si$	512.4044	497.3807, 482.3931, 422.3548, **409.3468**, 391.3361, 229.1951, 203.1792, 189.1638, 175.1481, 163.1480, 147.1168, 133.1011, 119.0855, 105.1698, 95.0855, 81.0698, 73.0468, 69.0698, 55.0542
η	Triterpenoid	44.34	$C_{39}H_{72}O_4Si_3$	688.4733	673.4502, 586.4599, 570.4295, 496.4104, 481.3865, 391.3365, **216.1873**, 203.1794, 189.1636, 175.1479, 161.1325, 143.1062, 129.0855, 107.0854, 95.0855, 81.0698, 73.0467, 69.0698, 55.0542
θ	Triterpenoid	48.00	$C_{33}H_{52}O_3Si$	524.3686	509.3446, 419.2949, 391.2991, **216.1508**, 203.1790, 189.1636, 171.1169, 159.1167, 145.1012, 133.1010, 119.0854, 109.1010, 95.0854, 81.0698, 73.0467, 67.0541, 55.0542
ι	Triterpenoid	48.36	$C_{33}H_{52}O_3Si$	524.3686	509.3452, 434.3177, 419.2947, 406.3233, 391.2994, 378.2919, 337.2521, **309.2214**, 269.1899, 244.1819, 229.1587, 185.1322, 169.0678, 157.1017, 143.0865, 133.1010, 119.0854, 107.0854, 95.0854, 81.0698, 73.0467, 67.0542, 55.0542
λ	Triterpenoid	49.16	$C_{36}H_{64}O_3Si_2$	600.4394	585.4167, 511.3605, **495.3661**, 421.3100, 405.3154, 377.3205, 295.2418, 241.1949, 227.1793, 189.1638, 169.0680, 159.1168, 145.1012, 135.1166, 119.0854, 107.0854, 95.0854, 81.0698, 73.0467, 64.0542, 55.0542

* the dppm between calculated and experimental masses was below 2 ppm in all cases. Base peak in bold.

References

1. Pachi, V.K., et al., *Chemical Profiling of Pistacia lentiscus var. Chia Resin and Essential Oil: Ageing Markers and Antimicrobial Activity.* Processes, 2021. **9**(3): p. 418.
2. Tabanca, N., et al., *Chemical Characterization and Biological Activity of the Mastic Gum Essential Oils of Pistacia lentiscus var. chia from Turkey.* Molecules, 2020. **25**(9): p. 2136.
3. Zachariadis, G.A. and A.V. Langioli, *Headspace Solid Phase Microextraction for Terpenes and Volatile Compounds Determination in Mastic Gum Extracts, Mastic Oil and Human Urine by GC– MS.* Analytical Letters, 2012. **45**(9): p. 993-1003.
4. Lo Presti, M., et al., *Evaluation of the volatile and chiral composition in Pistacia lentiscus L. essential oil.* Flavour and Fragrance Journal, 2008. **23**(4): p. 249-257.
5. Rageot, M., et al., *Biomolecular analyses enable new insights into ancient Egyptian embalming.* Nature, 2023. **614**(7947): p. 287-293.

Table S2. Summary of the results obtained for the samples taken from varnishes, coatings and residues on Egyptian coffins in the collection of the Fitzwilliam Museum. DAx = aliphatic dicarboxylic acids with x carbon atoms; Fx = aliphatic monocarboxylic acids with x carbon atoms. Triterpenoid numbers refer to Table 2.

Object no.	Sample	Description	Compounds identified
E.64.1896	F01A	Varnish on surface of Nakhtefmut cartonnage	Triterpenoids: 1; 2; 16; 17; 18<br>Oil/fat lipids: DA7; DA8; DA9; DA10; F16; F18.
E.1.1822	F09A<br>F09B<br>F09C<br>F09D<br>F09E<br>F09F<br>F09G<br>F09H<br>F09K	Yellow resin from various areas of Nespawershefyt coffin (lid and base both outer and inner coffin) and mummy board	Triterpenoids: 1; 2; 16; 17; 18<br>Oil/fat lipids: DA8; DA9; F16; F18; F18:1
E.GA.507.1947	F15A	Yellow varnish from coffin (face)	Triterpenoids: 1; 2; 16; 17; 18<br>Oil/fat lipids: DA7; DA8; DA9; DA10; F16; F18.<br>Diterpenoids: 7-oxo-DHA; 7-oxo-hydroxy-DHA
E.GA.528.1947	F16A	Yellow varnish from the surface of a coffin fragment	Triterpenoids: 1; 2; 16; 17; 18.<br>Oil/fat lipids: DA9 (tr); F16
E.GA.504.1947	F17A	Black resin from coffin (face)	Triterpenoids: 1; 2; 16; 17; 18<br>Oil/fat lipids: F14; F16; F18; F18:1.
E.GA.2672.1943	F18A	Yellow varnish from a coffin fragment	Triterpenoids: 1; 2; 16; 17; 18<br>Oil/fat lipids: DA8; DA9; DA10; F14; F16; F18; F18:1
E.558.1939	F20A	Yellow varnish from wooden ear (fragment)	Triterpenoids: 1; 2; 16; 17; 18<br>Oil/fat lipids: DA8; DA9; F16; F18.
E.GA.6548.1943	F21A	Yellow varnish from cartonnage fragment	Triterpenoids: 1; 2; 16; 17; 18<br>Oil/fat lipids: nd
E.GA.2888.1943	F23A	Yellow varnish from cartonnage fragment (below wing)	Triterpenoids: 1; 2; 16; 17; 18<br>Oil/fat lipids: DA8; DA9; F14; F16; F18;
E.GA.5851.1943	F26A	Black resin from wooden lotus (coffin fragment)	Triterpenoids: 1; 2; 16; 17; 18<br>Oil/fat lipids: DA9; F16; F18;
E.GA.2891.1943	F27A	Golden resin from front of cartonnage fragment	Triterpenoids: 1; 2; 16; 17; 18<br>Oil/fat lipids: F14; F16; F18; F18:1

E.GA.1174.1947	F28A	Golden resin from cartonnage fragment (chin)	Triterpenoids: 1; 2; 16; 17; 18<br>Oil/fat lipids: DA7; DA8; DA9; DA10; F16; F18; F18:1<br>Diterpenoids: DHA; 7-oxo-DHA; 7-oxo-hydroxy-DHA
E.GA.2870.1943	F30A	Yellow varnish from hand with Djed pillar	Triterpenoids: 1; 2; 14;15; 16; 17; 18; 24; 25 and others<br>Oil/fat lipids: nd.
EGA.503.1947	F37A	Black resin from coffin face	Triterpenoids: 1; 2; 14,15,16; 17; 18, 19, 20 and others<br>Oil/fat lipids: DA8; DA9; F16; F18;<br>Diterpenoids: retene; DHA; 7-oxo-DHA; 7-oxo-hydroxy-DHA and others
E.200.1939	F43A	Black resin from coffin face (wig)	Triterpenoids: 1; 2; 16; 17; 18<br>Oil/fat lipids: DA8; DA9; DA10; F16; F18; F24; F26<br>*n*-Alkanes C25-C31
E.114.1903	F45A	Detached fragment of amber coloured resin from Abydos	Triterpenoids: 1; 2; 6; 13; 14; 15; 17; 18; 19; 20; 24; 25; 33<br>Oil/fat lipids: DA9 (tr); F16; F18; F18:1
E.133.1891	F46A	Black resin from cartonnage fragment	Triterpenoids: 17; 18<br>Oil/fat lipids: DA7; DA8; DA9; F14; F16; F18; F24; F26: F28; F30<br>*n*-Alkanes C25-C33
E.W.94	F52B	Reddish resin from ‘loop’ of resin on back leg of jackal figure	Triterpenoids: 2; 16; 17; 18<br>Oil/fat lipids: DA8; F14; F16; F18;<br>Diterpenoids: DHA;
